# Heterogeneity in transmissibility and shedding SARS-CoV-2 via droplets and aerosols

**DOI:** 10.7554/eLife.65774

**Published:** 2021-04-16

**Authors:** Paul Z Chen, Niklas Bobrovitz, Zahra Premji, Marion Koopmans, David N Fisman, Frank X Gu

**Affiliations:** 1 Department of Chemical Engineering & Applied Chemistry, University of TorontoTorontoCanada; 2 Temerty Faculty of Medicine, University of TorontoTorontoCanada; 3 Department of Critical Care Medicine, Cumming School of Medicine, University of CalgaryCalgaryCanada; 4 O'Brien Institute of Public Health, University of CalgaryCalgaryCanada; 5 Libraries & Cultural Resources, University of CalgaryCalgaryCanada; 6 Department of Viroscience, Erasmus University Medical CenterRotterdamNetherlands; 7 Division of Epidemiology, Dalla Lana School of Public Health, University of TorontoTorontoCanada; 8 Division of Infectious Diseases, Temerty Faculty of Medicine, University of TorontoTorontoCanada; 9 Institute of Biomedical Engineering, University of TorontoTorontoCanada; Radboud University Medical CentreNetherlands; Radboud University Medical CentreNetherlands

**Keywords:** COVID-19, emerging pathogens, influenza, overdispersion, superspreading, None

## Abstract

**Background:**

Which virological factors mediate overdispersion in the transmissibility of emerging viruses remains a long-standing question in infectious disease epidemiology.

**Methods:**

Here, we use systematic review to develop a comprehensive dataset of respiratory viral loads (rVLs) of SARS-CoV-2, SARS-CoV-1 and influenza A(H1N1)pdm09. We then comparatively meta-analyze the data and model individual infectiousness by shedding viable virus via respiratory droplets and aerosols.

**Results:**

The analyses indicate heterogeneity in rVL as an intrinsic virological factor facilitating greater overdispersion for SARS-CoV-2 in the COVID-19 pandemic than A(H1N1)pdm09 in the 2009 influenza pandemic. For COVID-19, case heterogeneity remains broad throughout the infectious period, including for pediatric and asymptomatic infections. Hence, many COVID-19 cases inherently present minimal transmission risk, whereas highly infectious individuals shed tens to thousands of SARS-CoV-2 virions/min via droplets and aerosols while breathing, talking and singing. Coughing increases the contagiousness, especially in close contact, of symptomatic cases relative to asymptomatic ones. Infectiousness tends to be elevated between 1 and 5 days post-symptom onset.

**Conclusions:**

Intrinsic case variation in rVL facilitates overdispersion in the transmissibility of emerging respiratory viruses. Our findings present considerations for disease control in the COVID-19 pandemic as well as future outbreaks of novel viruses.

**Funding:**

Natural Sciences and Engineering Research Council of Canada (NSERC) Discovery Grant program, NSERC Senior Industrial Research Chair program and the Toronto COVID-19 Action Fund.

## eLife digest

To understand how viruses spread scientists look at two things. One is – on average – how many other people each infected person spreads the virus to. The other is how much variability there is in the number of people each person with the virus infects. Some viruses like the 2009 influenza H1N1, a new strain of influenza that caused a pandemic beginning in 2009, spread pretty uniformly, with many people with the virus infecting around two other people. Other viruses like SARS-CoV-2, the one that causes COVID-19, are more variable. About 10 to 20% of people with COVID-19 cause 80% of subsequent infections – which may lead to so-called superspreading events – while 60-75% of people with COVID-19 infect no one else.

Learning more about these differences can help public health officials create better ways to curb the spread of the virus. Chen et al. show that differences in the concentration of virus particles in the respiratory tract may help to explain why superspreaders play such a big role in transmitting SARS-CoV-2, but not the 2009 influenza H1N1 virus. Chen et al. reviewed and extracted data from studies that have collected how much virus is present in people infected with either SARS-CoV-2, a similar virus called SARS-CoV-1 that caused the SARS outbreak in 2003, or with 2009 influenza H1N1.

Chen et al. found that as the variability in the concentration of the virus in the airways increased, so did the variability in the number of people each person with the virus infects. Chen et al. further used mathematical models to estimate how many virus particles individuals with each infection would expel via droplets or aerosols, based on the differences in virus concentrations from their analyses. The models showed that most people with COVID-19 infect no one because they expel little – if any – infectious SARS-CoV-2 when they talk, breathe, sing or cough. Highly infectious individuals on the other hand have high concentrations of the virus in their airways, particularly the first few days after developing symptoms, and can expel tens to thousands of infectious virus particles per minute. By contrast, a greater proportion of people with 2009 influenza H1N1 were potentially infectious but tended to expel relatively little infectious virus when the talk, sing, breathe or cough.

These results help explain why superspreaders play such a key role in the ongoing pandemic. This information suggests that to stop this virus from spreading it is important to limit crowd sizes, shorten the duration of visits or gatherings, maintain social distancing, talk in low volumes around others, wear masks, and hold gatherings in well-ventilated settings. In addition, contact tracing can prioritize the contacts of people with high concentrations of virus in their airways.

## Introduction

Severe acute respiratory syndrome coronavirus 2 (SARS-CoV-2) has spread globally, causing the coronavirus disease 2019 (COVID-19) pandemic with more than 129.2 million infections and 2.8 million deaths (as of 1 April 2021) (Dong et al., 2020). For respiratory virus transmission, airway epithelial cells shed virions to the extracellular fluid before atomization (from breathing, talking, singing, coughing and aerosol-generating procedures) partitions them into a polydisperse mixture of particles that are expelled to the ambient environment. Aerosols (≤100 μm) can be inhaled nasally, whereas droplets (>100 μm) tend to be excluded (Prather et al., 2020; Roy and Milton, 2004). For direct transmission, droplets must be sprayed ballistically onto susceptible tissue (Liu et al., 2017a). Hence, droplets predominantly deposit on nearby surfaces, potentiating indirect transmission. Aerosols can be further categorized based on typical travel characteristics: short-range aerosols (50–100 μm) tend to settle within 2 m; long-range ones (10–50 μm) often travel beyond 2 m based on emission force; and buoyant aerosols (≤10 μm) remain suspended and travel based on airflow profiles for minutes to many hours (Liu et al., 2017a; Wei and Li, 2015). Although proximity has been associated with infection risk for COVID-19 (Chu et al., 2020), studies have also suggested that long-range airborne transmission occurs conditionally (Hamner et al., 2020; Lu et al., 2020a; Park et al., 2020).

While the basic reproductive number has been estimated to be 2.0–3.6 (Hao et al., 2020; Li et al., 2020a), transmissibility of SARS-CoV-2 is highly overdispersed (dispersion parameter *k*, 0.10–0.58), with numerous instances of superspreading (Hamner et al., 2020; Lu et al., 2020a; Park et al., 2020) and few cases (10–20%) causing many secondary infections (80%) (Bi et al., 2020; Endo et al., 2020; Laxminarayan et al., 2020). Similarly, few cases drive the transmission of SARS-CoV-1 (*k*, 0.16–0.17) (Lloyd-Smith et al., 2005), whereas influenza A(H1N1)pdm09 transmits more homogeneously (*k*, 7.4–14.4) (Brugger and Althaus, 2020; Roberts and Nishiura, 2011), despite both viruses spreading by contact, droplets and aerosols (Cowling et al., 2013; Yu et al., 2004). Although understanding the determinants of viral overdispersion is crucial towards characterizing transmissibility and developing effective strategies to limit infection (Lee et al., 2020), mechanistic associations for *k* remain unclear. As an empirical estimate, *k* depends on myriad extrinsic (behavioral, environmental and invention) and host factors. Nonetheless, *k* remains similar across distinct outbreaks for a virus (Lloyd-Smith et al., 2005), suggesting that intrinsic virological factors mediate virus overdispersion.

Here, we investigated how intrinsic case variation in respiratory viral loads (rVLs) facilitates overdispersion in SARS-CoV-2 transmissibility. By systematic review, we developed a comprehensive dataset of rVLs from cases of COVID-19, SARS and A(H1N1)pdm09. Using comparative meta-analyses, we found that heterogeneity in rVL was associated with overdispersion among these emerging infections. To assess potential sources of case heterogeneity, we analyzed SARS-CoV-2 rVLs across age and symptomatology subgroups as well as disease course. To interpret the influence of heterogeneity in rVL on individual infectiousness, we modeled likelihoods of shedding viable virus via respiratory droplets and aerosols.

## Results

### Systematic review

**Figure 1.**
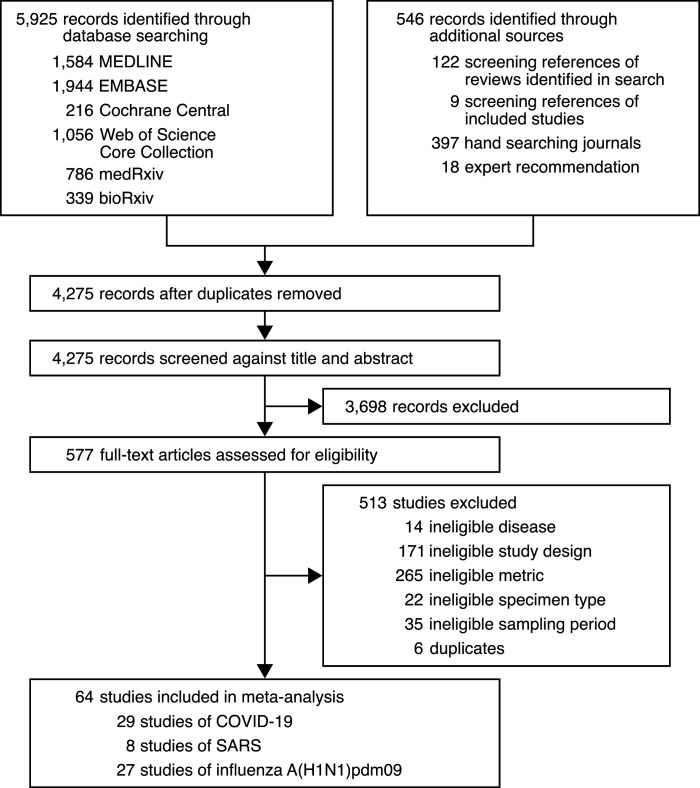
Development of the systematic dataset. Figure 1—source data 1.Search strategy used for MEDLINE. Figure 1—source data 2.Search strategy used for EMBASE. Figure 1—source data 3.Search strategy used for Cochrane Central. Figure 1—source data 4.Search strategy used for Web of Science Core Collection. Figure 1—source data 5.Search strategy used for medRxiv and bioRxiv.

We conducted a systematic review on virus quantitation in respiratory specimens taken during the infectious periods of SARS-CoV-2 (−3 to 10 days from symptom onset [DFSO]) (Arons et al., 2020; He et al., 2020; Wölfel et al., 2020), SARS-CoV-1 (0–20 DFSO) (Pitzer et al., 2007) and A(H1N1)pdm09 (−2 to 9 DFSO) (Ip et al., 2017) (Materials and methods). The systematic search (Figure 1—source data 1, Figure 1—source data 2, Figure 1—source data 3, Figure 1—source data 4, Figure 1—source data 5) identified 4274 results. After screening and full-text review, 64 studies met the inclusion criteria and contributed to the systematic dataset (Figure 1) (*N* = 9631 total specimens), which included adult (*N* = 5033) and pediatric (*N* = 1608) cases from 15 countries and specimen measurements for asymptomatic (*N* = 2387), presymptomatic (*N* = 28) and symptomatic (*N* = 7161) infections. According to a hybrid Joanna Briggs Institute critical appraisal checklist, risk of bias was low for most contributing studies (Appendix 1—table 1).

### Association of overdispersion with heterogeneity in rVL

We hypothesized that individual case variation in rVL facilitates the distinctions in *k* among COVID-19, SARS and A(H1N1)pdm09. For each study in the systematic dataset, we used specimen measurements (viral RNA concentration in a respiratory specimen) to estimate rVLs (viral RNA concentration in the respiratory tract) (Materials and methods). To investigate the relationship between *k* and heterogeneity in rVL, we performed a meta-regression using each contributing study (Figure 2—figure supplement 1), which showed a weak, negative association between the two variables (meta-regression slope *t*-test: p=0.038, Pearson’s *r* = −0.26).

**Figure 2.**
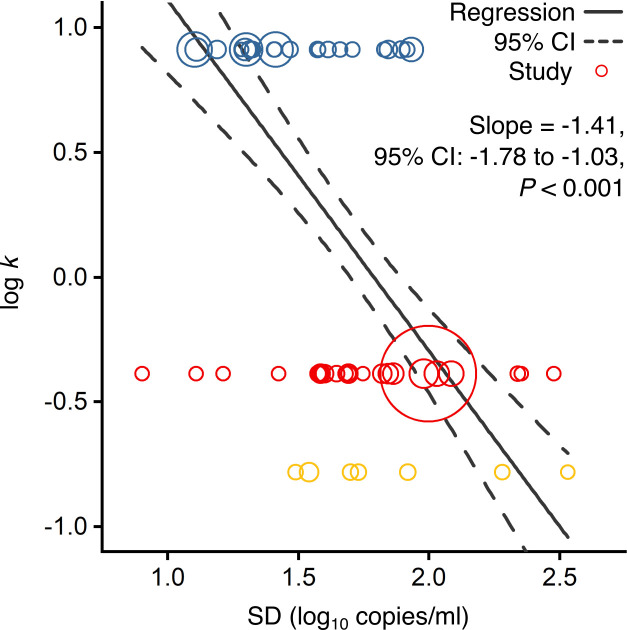
Association of overdispersion in SARS-CoV-2, SARS-CoV-1 and A(H1N1)pdm09 transmissibility with heterogeneity in respiratory viral load (rVL). Meta-regression of dispersion parameter (*k*) with the standard deviation (SD) of rVLs from contributing studies with low risk of bias (Pearson’s *r* = −0.73). Pooled estimates of *k* were determined from the literature for each infection. Blue, red and yellow circles denote A(H1N1)pdm09 (*N* = 22), COVID-19 (*N* = 24) and SARS (*N* = 7) studies, respectively. Circle sizes denote weighting in the meta-regression. The p-value was obtained using the meta-regression slope *t*-test.

Using contributing studies with low risk of bias, meta-regression (Figure 2) showed a strong, negative association between *k* and heterogeneity in rVL for these three viruses (meta-regression slope *t*-test: p<0.001, Pearson’s *r* = −0.73). In this case, each unit increase (one log_10_ copies/ml) in the standard deviation (SD) of rVL decreased log(*k*) by a factor of −1.41 (95% confidence interval [CI]: −1.78 to −1.03), suggesting that broader heterogeneity in rVL facilitates greater overdispersion in the transmissibility of SARS-CoV-2 than of A(H1N1)pmd09. To investigate mechanistic aspects of this association, we conducted a series of analyses on rVL and then modeled the influence of heterogeneity in rVL on individual infectiousness.

**Figure 2—figure supplement 1.**
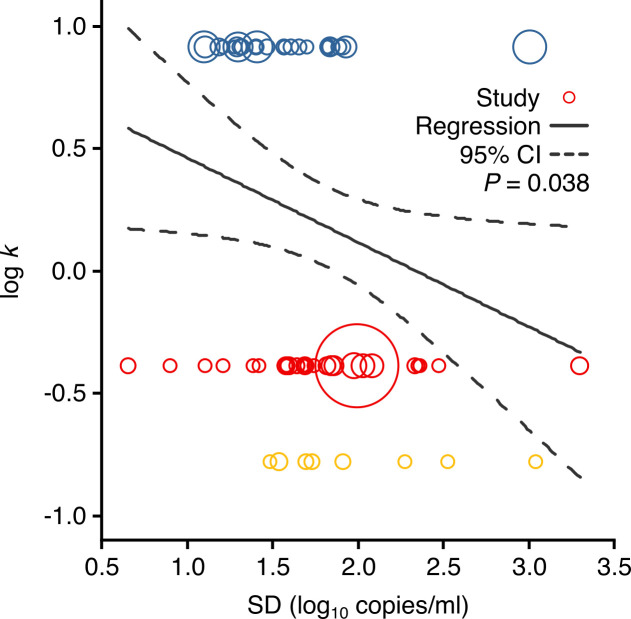
Meta-regression between dispersion in SARS-CoV-2, SARS-CoV-1 and A(H1N1)pdm09 transmissibility and heterogeneity in respiratory viral load (rVL). Meta-regression of dispersion parameter (*k*) with the standard deviation (SD) of rVLs from all contributing studies (Pearson’s *r* = −0.26). Pooled estimates of *k* were determined from the literature. Blue, red and yellow circles denote A(H1N1)pdm09 (*N* = 27), COVID-19 (*N* = 29) and SARS (*N* = 8) studies, respectively. Circle sizes denote weighting in the meta-regression. The p-value was obtained using the meta-regression slope *t*-test.

**Figure 2—figure supplement 2.**
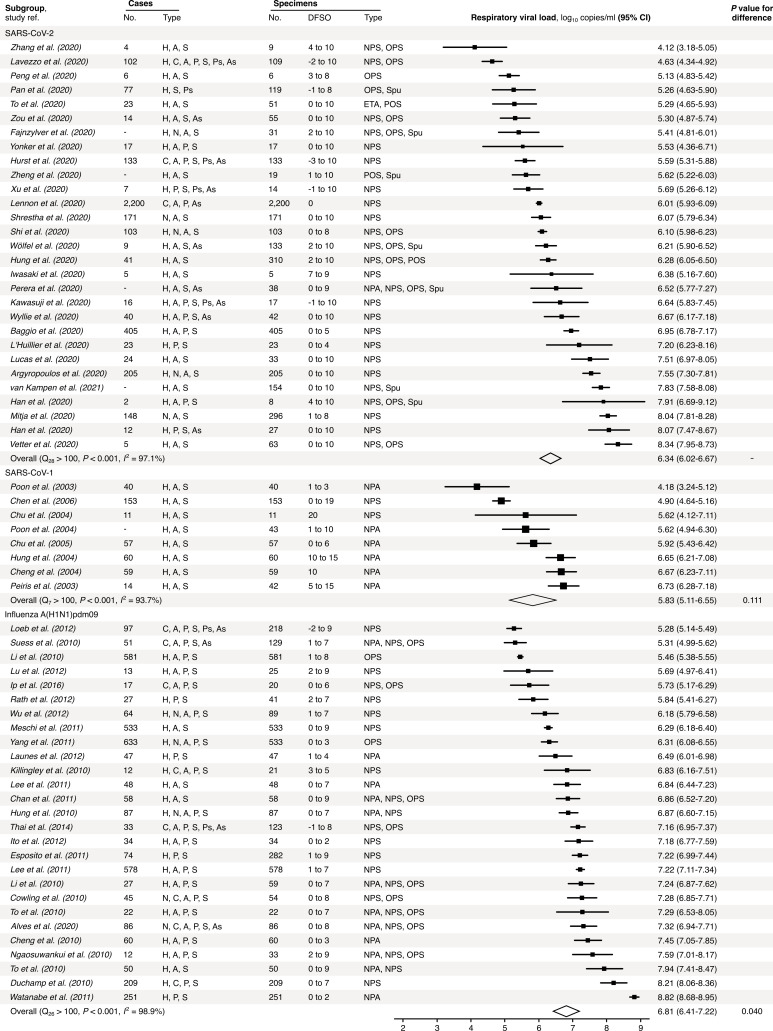
Meta-analysis of respiratory viral loads (rVLs) of SARS-CoV-2, SARS-CoV-1 and influenza A(H1N1)pdm09 during the estimated infectious period. Random-effects meta-analyses comparing the expected rVLs for COVID-19, SARS and A(H1N1)pdm09 cases during the infectious period. Quantitative specimen measurements were used to estimate rVLs, which refer to virus concentrations in the respiratory tract. Case types: hospitalized (H), not admitted (N), community (C), adult (A), pediatric (P), symptomatic (S), presymptomatic (Ps) and asymptomatic (As). Specimen types: endotracheal aspirate (ETA), nasopharyngeal aspirate (NPA), nasopharyngeal swab (NPS), oropharyngeal swab (OPS), posterior oropharyngeal saliva (POS) and sputum (Spu). Dashes denote case numbers that were not obtained. Box sizes denote weighting in the overall estimates. Between-study heterogeneity was assessed using the p-value from Cochran’s *Q* test and the *I*^2^ statistic. One-sided Welch’s *t*-tests compared the expected SARS-CoV-2 rVL with those of SARS-CoV-1 and A(H1N1)pdm09 (non-significance, p>0.05).

### Meta-analysis and subgroup analyses of rVL

We first compared rVLs among the emerging infections. We performed a random-effects meta-analysis (Figure 2—figure supplement 2), which approximated the expected rVL when encountering a COVID-19, SARS or A(H1N1)pdm09 case during the infectious period. This showed that the expected rVL of SARS-CoV-2 was comparable to that of SARS-CoV-1 (one-sided Welch’s *t*-test: p=0.111) but lesser than that of A(H1N1)pdm09 (p=0.040).

**Figure 3.**
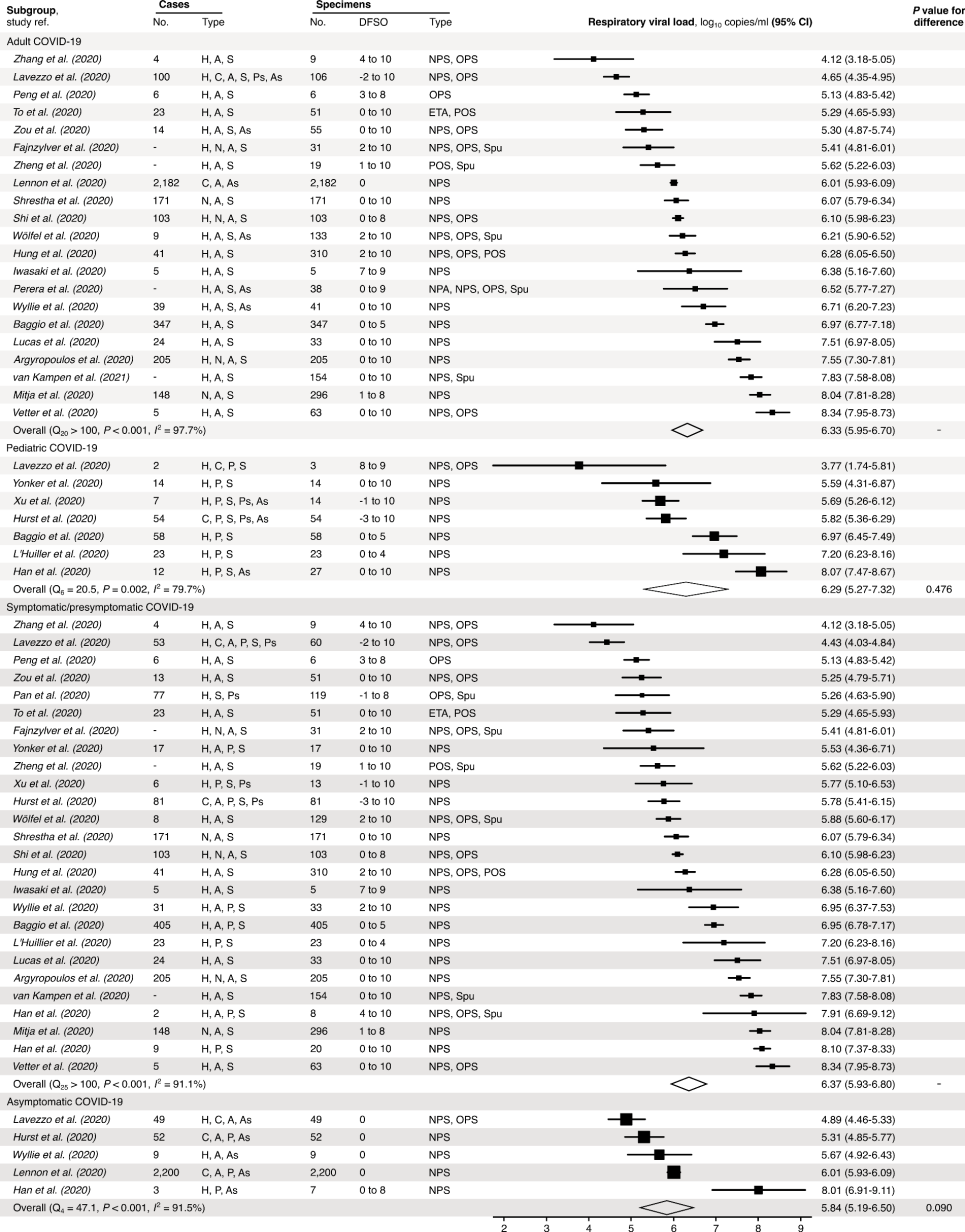
Subgroup analyses of SARS-CoV-2 respiratory viral load (rVL) during the infectious period. Random-effects meta-analyses comparing the expected rVLs of adult (≥18 years old) COVID-19 cases with pediatric (<18 years old) ones (top) and symptomatic/presymptomatic infections with asymptomatic ones (bottom) during the infectious period. Quantitative rVLs refer to virus concentrations in the respiratory tract. Case types: hospitalized (H), not admitted (N), community (C), adult (A), pediatric (P), symptomatic (S), presymptomatic (Ps) and asymptomatic (As). Specimen types: endotracheal aspirate (ETA), nasopharyngeal aspirate (NPA), nasopharyngeal swab (NPS), oropharyngeal swab (OPS), posterior oropharyngeal saliva (POS) and sputum (Spu). Dashes denote case numbers that were not obtained. Box sizes denote weighting in the overall estimates. Between-study heterogeneity was assessed using the p-value from Cochran’s *Q* test and the *I*^2^ statistic. One-sided Welch’s *t*-tests compared expected rVLs between the COVID-19 subgroups (non-significance, p>0.05).

We also performed random-effects subgroup analyses for COVID-19 (Figure 3), which showed that expected SARS-CoV-2 rVLs were similar between pediatric and adult cases (p=0.476) and comparable between symptomatic/presymptomatic and asymptomatic infections (p=0.090). Since these meta-analyses had significant between-study heterogeneity among the mean estimates (Cochran’s *Q* test: p<0.001 for each meta-analysis), we conducted risk-of-bias sensitivity analyses; meta-analyses of low-risk-of-bias studies continued to show significant heterogeneity (Figure 3—figure supplements 1–5).

**Figure 3—figure supplement 1.**
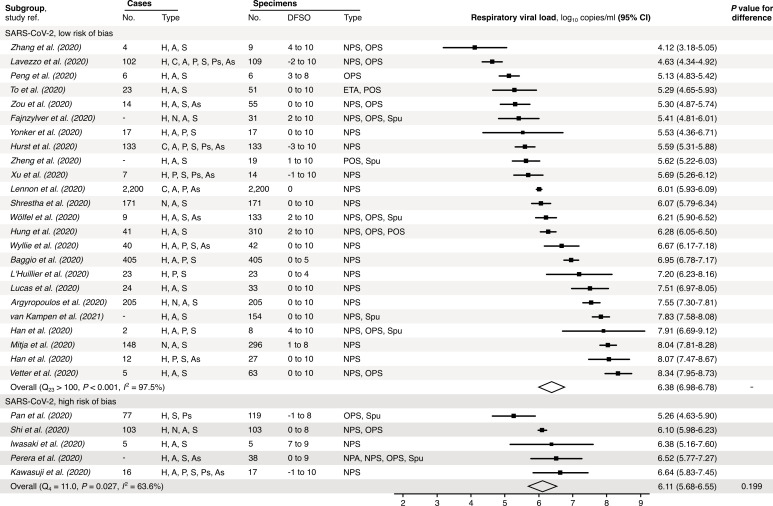
Risk-of-bias sensitivity analysis of between-study heterogeneity for SARS-CoV-2 respiratory viral load (rVL) during the estimated infectious period. Random-effects meta-analyses, based on the risk of bias of contributing studies, of the expected rVLs of COVID-19 cases during the infectious period. Quantitative rVLs refer to virus concentrations in the respiratory tract. Case types: hospitalized (H), not admitted (N), community (C), adult (A), pediatric (P), symptomatic (S), presymptomatic (Ps) and asymptomatic (As). Specimen types: endotracheal aspirate (ETA), nasopharyngeal aspirate (NPA), nasopharyngeal swab (NPS), oropharyngeal swab (OPS), posterior oropharyngeal saliva (POS) and sputum (Spu). Dashes denote case numbers that were not obtained. Box sizes denote weighting in the overall estimates. One-sided Welch’s *t*-test for difference (non-significance, p>0.05). Between-study heterogeneity was assessed using the p-value from Cochran’s *Q* test (non-significance, p>0.05) and the *I*^2^ statistic (*I*^2^ < 30% indicates low between-study heterogeneity).

**Figure 3—figure supplement 2.**
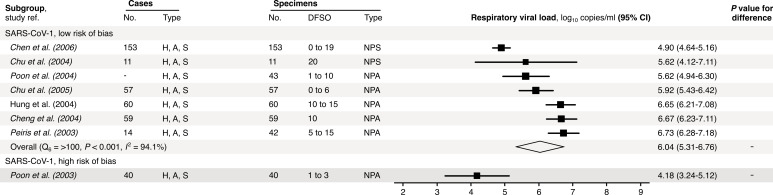
Risk-of-bias sensitivity analysis of between-study heterogeneity for SARS-CoV-1 respiratory viral load (rVL) during the estimated infectious period. Random-effects meta-analyses, based on the risk of bias of contributing studies, of the expected rVLs of SARS cases during the infectious period. Quantitative rVLs refer to virus concentrations in the respiratory tract. Case types: hospitalized (H), adult (A) and symptomatic (S). Specimen types: nasopharyngeal aspirate (NPA) and nasopharyngeal swab (NPS). Dashes denote case numbers that were not obtained. Box sizes denote weighting in the overall estimates. One-sided Welch’s *t*-test for difference, non-significance (p>0.05). Between-study heterogeneity was assessed using the p-value from Cochran’s *Q* test (non-significance, p>0.05) and the *I*^2^ statistic (*I*^2^ < 30% indicates low between-study heterogeneity).

**Figure 3—figure supplement 3.**
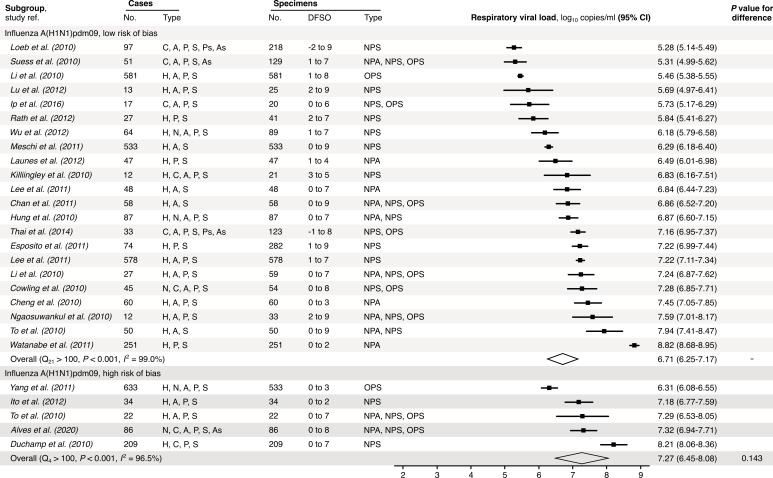
Risk-of-bias sensitivity analysis of between-study heterogeneity for A(H1N1)pdm09 respiratory viral load (rVL) during the estimated infectious period. Random-effects meta-analyses, based on the risk of bias of contributing studies, of the expected rVLs of A(H1N1)pdm09 cases during the infectious period. Quantitative rVLs refer to virus concentrations in the respiratory tract. Case types: hospitalized (H), not admitted (N), community (C), adult (A), pediatric (P), symptomatic (S), presymptomatic (Ps) and asymptomatic (As). Specimen types: nasopharyngeal aspirate (NPA), nasopharyngeal swab (NPS) and oropharyngeal swab (OPS). Dashes denote case numbers that were not obtained. Box sizes denote weighting in the overall estimates. One-sided Welch’s *t*-test for difference (non-significance, p>0.05). Between-study heterogeneity was assessed using the p-value from Cochran’s *Q* test (non-significance, p>0.05) and the *I*^2^ statistic (*I*^2^ < 30% indicates low between-study heterogeneity).

**Figure 3—figure supplement 4.**
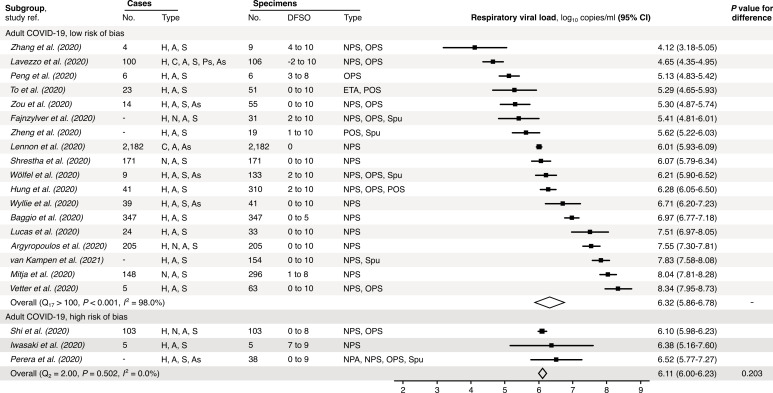
Risk-of-bias sensitivity analysis of between-study heterogeneity for SARS-CoV-2 respiratory viral load (rVL) for adult COVID-19 cases during the estimated infectious period. Random-effects meta-analyses, based on the risk of bias of contributing studies, of the expected rVLs of adult (≥18 years old) COVID-19 cases during the infectious period. Quantitative rVLs refer to virus concentrations in the respiratory tract. Case types: hospitalized (H), not admitted (N), community (C), adult (A), pediatric (P), symptomatic (S), presymptomatic (Ps) and asymptomatic (As). Specimen types: endotracheal aspirate (ETA), nasopharyngeal aspirate (NPA), nasopharyngeal swab (NPS), oropharyngeal swab (OPS), posterior oropharyngeal saliva (POS) and sputum (Spu). Dashes denote case numbers that were not obtained. Box sizes denote weighting in the overall estimates. One-sided Welch’s *t*-test for difference (non-significance, p>0.05). Between-study heterogeneity was assessed using the p-value from Cochran’s *Q* test (non-significance, p>0.05) and the *I*^2^ statistic (*I*^2^ <30% indicates low between-study heterogeneity).

**Figure 3—figure supplement 5.**
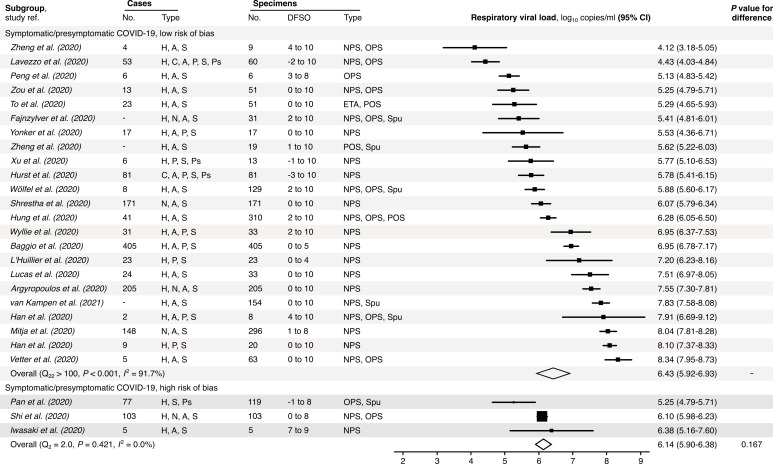
Risk-of-bias sensitivity analysis of between-study heterogeneity for SARS-CoV-2 respiratory viral load (rVL) for symptomatic/presymptomatic COVID-19 cases during the estimated infectious period. Random-effects meta-analyses, based on the risk of bias of contributing studies, of the expected rVLs of symptomatic/presymptomatic (≥18 years old) COVID-19 cases during the infectious period. Quantitative rVLs refer to virus concentrations in the respiratory tract. Case types: hospitalized (H), not admitted (N), community (C), adult (A), pediatric (P), symptomatic (S), presymptomatic (Ps) and asymptomatic (As). Specimen types: endotracheal aspirate (ETA), nasopharyngeal swab (NPS), oropharyngeal swab (OPS), posterior oropharyngeal saliva (POS) and sputum (Spu). Dashes denote case numbers that were not obtained. Box sizes denote weighting in the overall estimates. One-sided Welch’s *t*-test for difference (non-significance, p>0.05). Between-study heterogeneity was assessed using the p-value from Cochran’s *Q* test (non-significance, p>0.05) and the *I*^2^ statistic the *I*^2^ statistic (*I*^2^ < 30% indicates low between-study heterogeneity).

### Distributions of rVL

**Figure 4.**
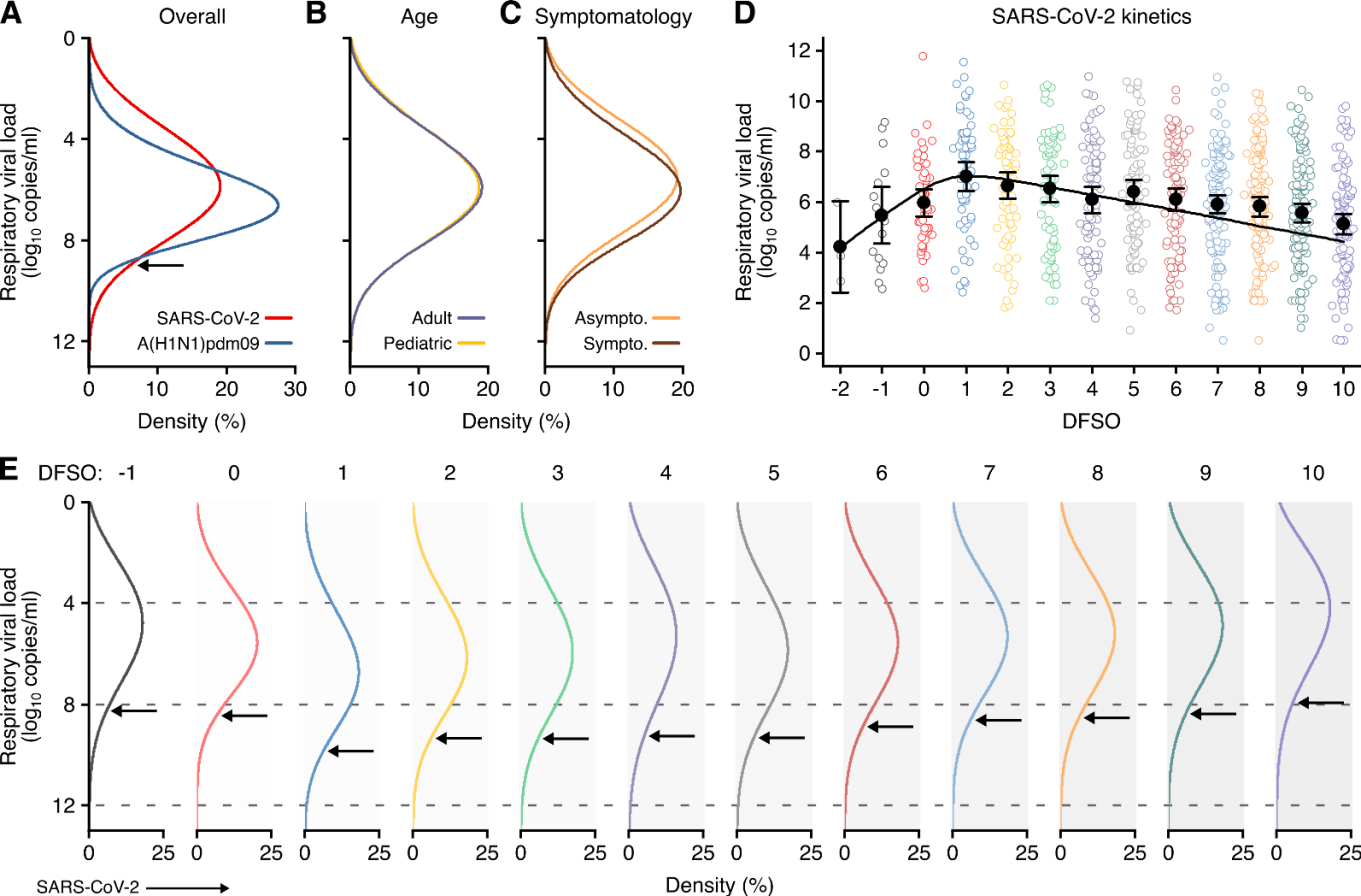
Heterogeneity and kinetics of SARS-CoV-2 respiratory viral load (rVL). (**A**) Estimated distribution of rVL for SARS-CoV-2 (*N* = 3834 samples from *N* = 26 studies) and A(H1N1)pdm09 (*N* = 512 samples from *N* = 10 studies) throughout the infectious periods. (**B, C**) Estimated distribution of SARS-CoV-2 rVL for adult (*N* = 3575 samples from *N* = 20 studies) and pediatric (*N* = 198 samples from *N* = 9 studies) (**B**) and symptomatic/presymptomatic (*N* = 1574 samples from *N* = 22 studies) and asymptomatic (*N* = 2221 samples from *N* = 7 studies) (**C**) COVID-19 cases. (**D**) SARS-CoV-2 rVLs fitted to a mechanistic model of viral kinetics (black curve, *r*^2^ = 0.84 for mean estimates). Filled circles and bars depict mean estimates and 95% confidence intervals. Open circles show the entirety of individual sample data over days from symptom onset (DFSO) (left to right*, N* = 3, 15, 50, 63, 71, 75, 85, 93, 105, 136, 123, 128 and 115 samples from *N* = 21 studies). (**E**) Estimated distributions of SARS-CoV-2 rVL across DFSO. Weibull distributions were fitted on the entirety of individual sample data for the virus, subgroup or DFSO in the systematic dataset. Arrows denote 90th case percentiles for SARS-CoV-2 rVL distributions.

We next analyzed rVL distributions. For all three viruses, rVLs best conformed to Weibull distributions (Figure 4—figure supplement 1), and we fitted the entirety of individual sample data for each virus in the systematic dataset (Figure 4A, Figure 4—figure supplement 1N). While COVID-19 and SARS cases tended to shed lesser virus than those with A(H1N1)pdm09 (Figure 2—figure supplement 2), broad heterogeneity in SARS-CoV-2 and SARS-CoV-1 rVLs inverted this relationship for highly infectious individuals (Figure 4A, Figure 4—figure supplement 2A-C). At the 90th case percentile (cp) throughout the infectious period, the estimated rVL was 8.91 (95% CI: 8.83–9.00) log_10_ copies/ml for SARS-CoV-2, whereas it was 8.62 (8.47–8.76) log_10_ copies/ml for A(H1N1)pdm09 (Figure 4—figure supplement 3). The SD of the overall rVL distribution for SARS-CoV-2 was 2.04 log_10_ copies/ml, while it was 1.45 log_10_ copies/ml for A(H1N1)pdm09, showing that heterogeneity in rVL was indeed broader for SARS-CoV-2.

**Figure 4—figure supplement 1.**
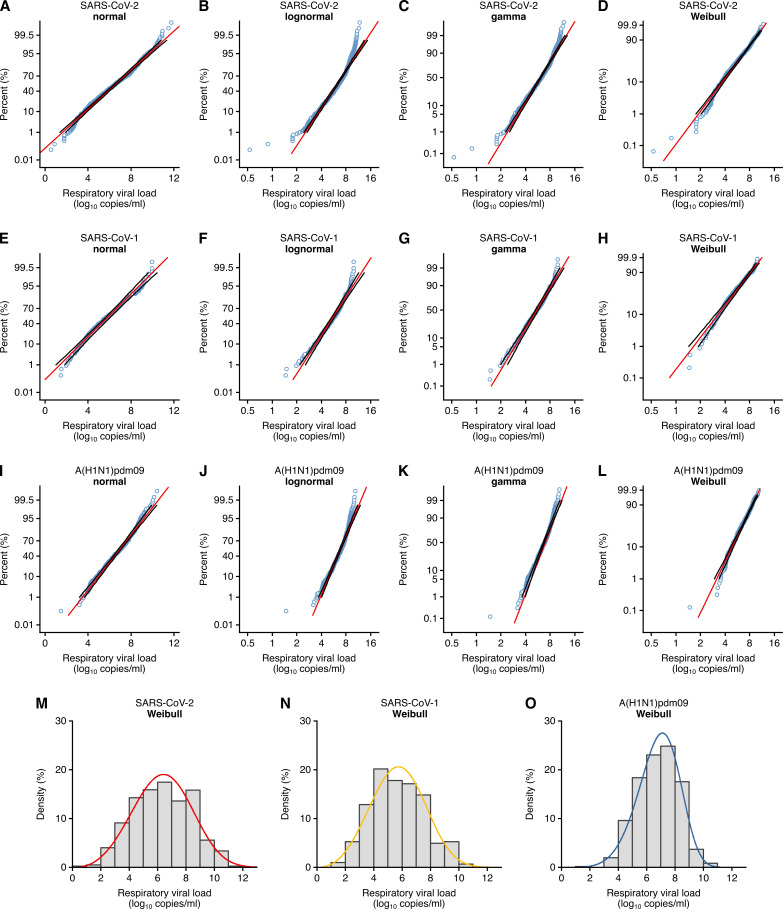
Respiratory viral loads for SARS-CoV-2, SARS-CoV-1 and A(H1N1)pdm09 best conform to Weibull distributions. (**A**–**D**) Normal (p≤0.01) (**A**), lognormal (p≤0.01) (**B**), gamma (p≤0.005) (**C**) and Weibull (p>0.10, not significant [NS]) (**D**) probability plots for individual sample data of SARS-CoV-2 respiratory viral loads (rVLs) across days from symptom onset in the systematic dataset (*N* = 941 samples from *N* = 20 studies). (**E**–**H**) Normal (p>0.05, NS) (**E**), lognormal (p≤0.01) (**F**), gamma (p>0.05, NS) (**G**) and Weibull (p>0.10, NS) (**H**) probability plots for individual sample data of SARS-CoV-1 rVLs in the systematic dataset (*N* = 303 samples from *N* = 5 studies). (**I**– **L**) Normal (p≤0.01) (**I**), lognormal (p≤0.01) (**J**), gamma (p≤0.005) (**K**) and Weibull (p>0.10, NS) (**L**) probability plots for individual sample data of A(H1N1)pdm09 rVLs in the systematic dataset (*N* = 512 samples from *N* = 10 studies). These categories included rVL data from positive (above the detection limit) assay measurements. The p-values were determined using the modified Kolmogorov–Smirnov test for the goodness of fit of each distribution. When the null hypothesis is accepted (NS at p>0.05), the probability density function cannot be rejected to describe the distribution of the data. Blue circles, black lines and red lines represent individual sample data, expected distributions and 95% confidence intervals, respectively. (**M**–**O**) Histograms and fitted Weibull distributions of the above data for SARS-CoV-2 (**M**), SARS-CoV-1 (**N**) and A(H1N1)pdm09 (**O**).

**Figure 4—figure supplement 2.**
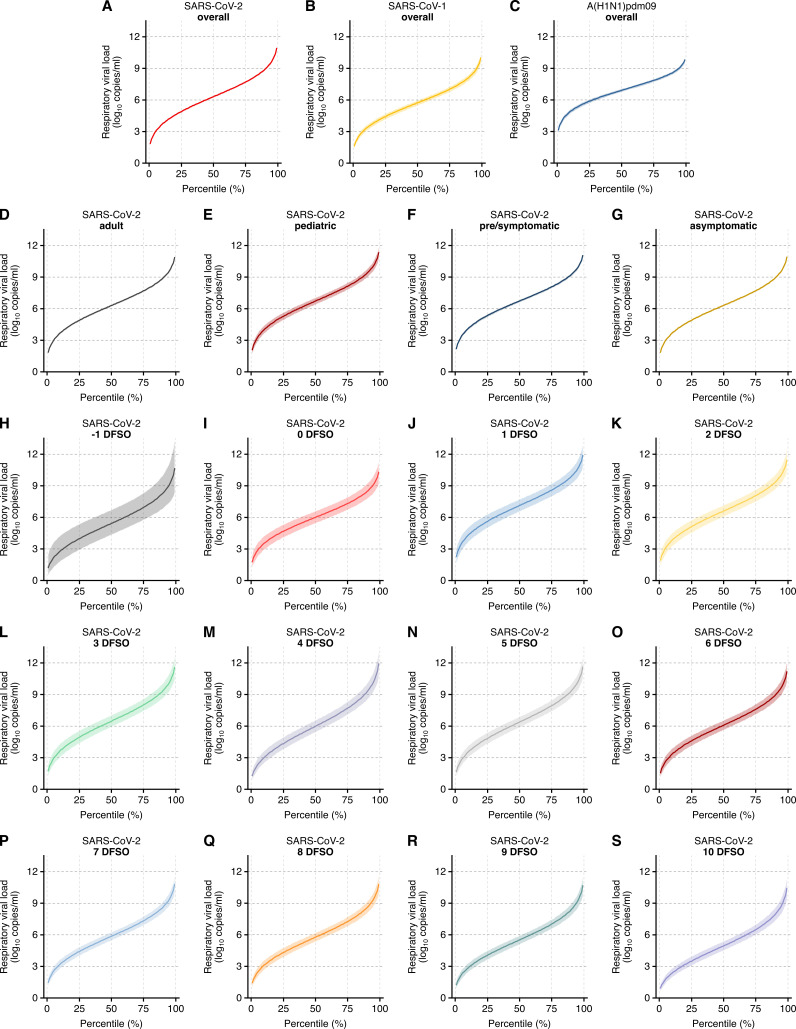
Case heterogeneity in respiratory viral loads (rVLs) across viruses, COVID-19 subgroups and disease course. (**A–C**) Estimated rVLs of SARS-CoV-2 (**A**), SARS-CoV-1 (**B**) and A(H1N1)pdm09 (**C**) across case percentile (cp) throughout the infectious periods. (**D**–**G**) Estimated SARS-CoV-2 rVLs for adult (**D**), pediatric (**E**), symptomatic/presymptomatic (**F**) and asymptomatic (**G**) cases across cp throughout the infectious period. (**H**–**S**) Estimated SARS-CoV-2 rVLs across cp on different days from symptom onset (DFSO) during the infectious period. Earlier DFSO were excluded based on limited data. Data ranged between the 1st and 99th cps. Lines and bands represent estimates and 95% confidence intervals, respectively.

**Figure 4—figure supplement 3.**
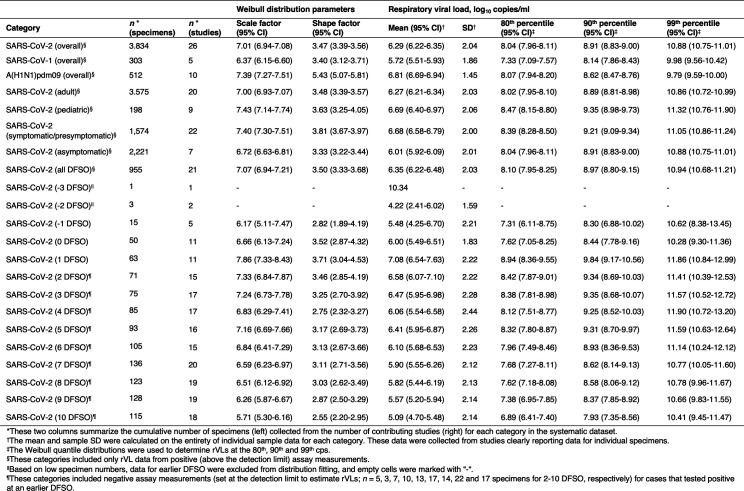
Descriptive parameters for respiratory viral loads based on individual sample data. Figure 4—figure supplement 3—source data 1.Editable version of Figure 4—figure supplement 3.

**Figure 4—figure supplement 4.**
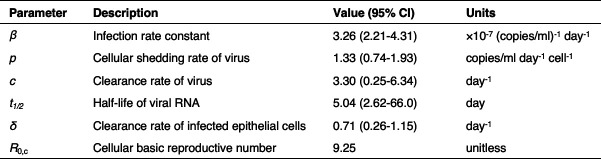
Model parameters describing SARS-CoV-2 kinetics during respiratory infection. Figure 4—figure supplement 4—source data 1.Editable version of Figure 4—figure supplement 4.

**Figure 4—figure supplement 5.**
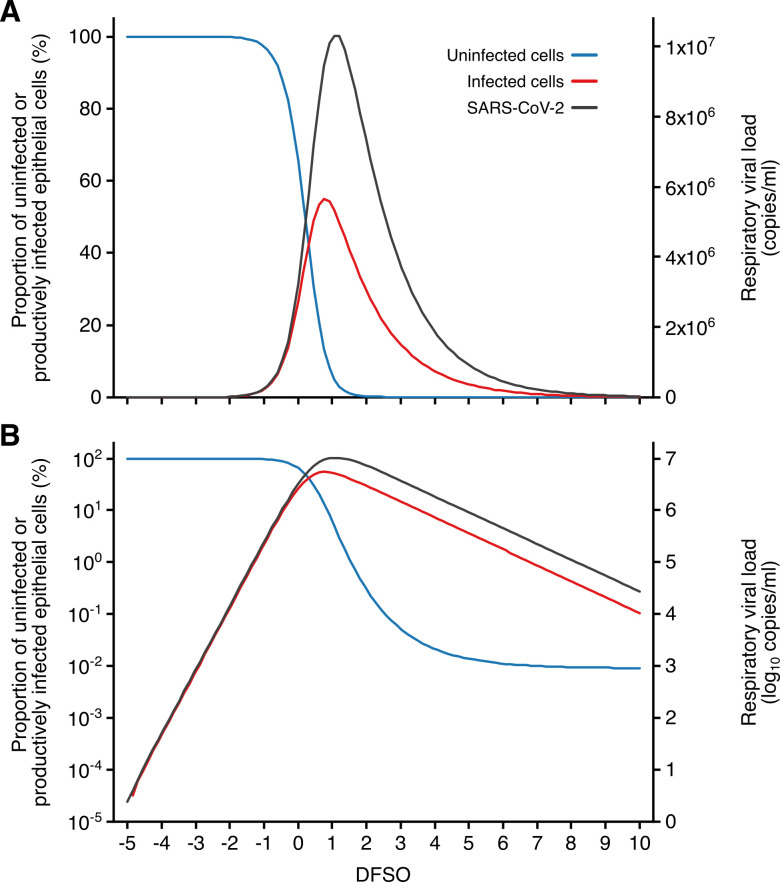
Kinetics of SARS-CoV-2 and airway epithelial cells during respiratory infection. (**A, B**) Estimated kinetics of uninfected (blue) and productively infected (red) airway epithelial cells (left axis) and SARS-CoV-2 (right axis) in the respiratory tract, as shown in linear (**A**) and logarithmic (**B**) scales.

To assess potential sources of heterogeneity in SARS-CoV-2 rVL, we compared rVL distributions among COVID-19 subgroups. In addition to comparable mean estimates (Figure 3), adult, pediatric, symptomatic/presymptomatic and asymptomatic COVID-19 cases showed similar rVL distributions (Figure 4B, C), with SDs of 2.03, 2.06, 2.00 and 2.01 log_10_ copies/ml, respectively. Thus, age and symptomatology minimally influenced case variation in SARS-CoV-2 rVL during the infectious period.

### SARS-CoV-2 kinetics during respiratory infection

To analyze the influences of disease course, we delineated individual SARS-CoV-2 rVLs by DFSO and fitted the mean estimates to a mechanistic model for respiratory virus kinetics (Figure 4D and Materials and methods). The outputs indicated that, on average, each productively infected cell in the airway epithelium shed SARS-CoV-2 at 1.33 (95% CI: 0.74–1.93) copies/ml day^−1^ and infected up to 9.25 susceptible cells (Figure 4—figure supplement 4). The turnover rate for infected epithelial cells was 0.71 (0.26–1.15) days^−1^, while the half-life of SARS-CoV-2 RNA before clearance from the respiratory tract was 0.21 (0.11–2.75) days. By extrapolating the model to an initial rVL of 0 log_10_ copies/ml, the estimated incubation period was 5.38 days, which agrees with epidemiological findings (Li et al., 2020a). Conversely, the expected duration of shedding was 25.1 DFSO. Thus, SARS-CoV-2 rVL increased exponentially after infection, peaked around 1 DFSO along with the proportion of infected epithelial cells (Figure 4—figure supplement 5) and then diminished exponentially.

To evaluate case heterogeneity across the infectious period, we fitted distributions for each DFSO (Figure 4E), which showed that high SARS-CoV-2 rVLs also increased from the presymptomatic period, peaked at 1 DFSO and then decreased towards the end of the first week of illness. For the 90th cp at 1 DFSO, the rVL was 9.84 (95% CI: 9.17–10.56) log_10_ copies/ml, an order of magnitude greater than the overall 90th cp estimate. High rVLs between 1 and 5 DFSO were elevated above the expected values from the overall rVL distribution (Figure 4—figure supplement 3). At −1 DFSO, the 90th cp rVL was 8.30 (6.88–10.02) log_10_ copies/ml, while it was 7.93 (7.35–8.56) log_10_ copies/ml at 10 DFSO. Moreover, heterogeneity in rVL remained broad across the infectious period, with SDs of 1.83–2.44 log_10_ copies/ml between −1 to 10 DFSO (Figure 4—figure supplement 2H-S).

### Likelihood that droplets and aerosols contain virions

Towards analyzing the influence of heterogeneity in rVL on individual infectiousness, we first modeled the likelihood of respiratory particles containing viable SARS-CoV-2. Since rVL is an intensive quantity, the volume fraction of virions is low and viral partitioning coincides with atomization, we used Poisson statistics to model likelihood profiles. To calculate an unbiased estimator of partitioning (the expected number of viable copies per particle), our method multiplied rVL estimates with particle volumes during atomization and an assumed viability proportion of 0.1% in equilibrated particles (Materials and methods).

When expelled by the mean COVID-19 case during the infectious period, respiratory particles showed low likelihoods of carrying viable SARS-CoV-2 (Figure 5—figure supplement 1). Aerosols (equilibrium aerodynamic diameter [*d*_a_] ≤ 100 µm) were ≤3.16% (95% CI: 2.61–3.71%) likely to contain a virion. Droplets also had low likelihoods: at *d*_a_ = 200 µm, they were 22.3% (21.4–23.2%), 3.36% (3.03–3.69%) and 0.34% (0.29–0.39%) likely to contain one, two or three virions, respectively.

**Figure 5.**
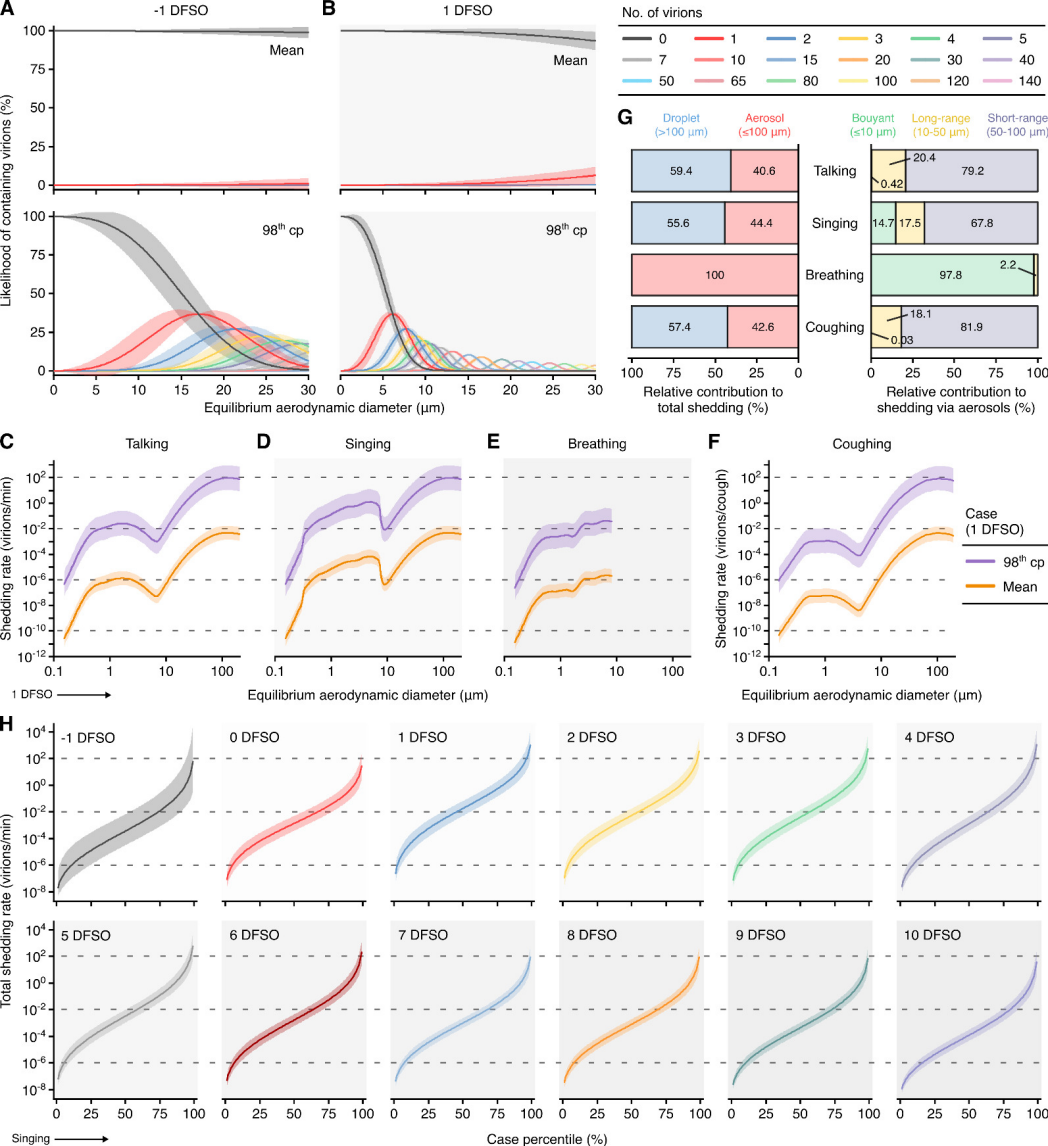
Heterogeneity in shedding SARS-CoV-2 via droplets and aerosols. (**A, B**) Estimated likelihood of respiratory particles containing viable SARS-CoV-2 when expelled by the mean (top) or 98th case percentile (cp) (bottom) COVID-19 cases at −1 (**A**) or 1 (**B**) days from symptom onset (DFSO). For higher number of virions, some likelihood curves were omitted to aid visualization. When the likelihood for zero virions approaches 0%, particles are expected to contain at least one viable copy. (**C**–**F**) Rate that the mean and 98th cp COVID-19 cases at 1 DFSO shed viable SARS-CoV-2 by talking, singing, breathing or coughing over particle size. (**G**) Relative contributions of droplets and aerosols to shedding virions for each respiratory activity (left). Relative contribution of buoyant, long-range and short-range aerosols to shedding virions via aerosols for each respiratory activity (right). (**H**) Case heterogeneity in the total shedding rate (over all particle sizes) of virions via singing across the infectious period. Earlier presymptomatic days were excluded based on limited data. Data range between the 1st and 99th cps. Lines and bands represent estimates and 95% confidence intervals, respectively, for estimated likelihoods or Poisson means.

COVID-19 cases with high rVLs, however, expelled particles with considerably greater likelihoods of carrying viable copies (Figure 5A, B, Figure 5—figure supplement 1D, E). For the 80th cp during the infectious period, aerosols (*d*_a_ ≤ 100 µm) were ≤87.9% (95% CI: 87.2–88.5%) likely to carry at least one SARS-CoV-2 virion. For the 90th cp, larger aerosols tended to contain multiple virions (Figure 5—figure supplement 1E). At 1 DFSO, these estimates were greatest, and ≤98.8% (98.1–99.4%) of buoyant aerosols (*d*_a_ ≤ 10 µm) contained at least one viable copy of SARS-CoV-2 for the 98th cp. When expelled by high cps, droplets (*d*_a_ > 100 µm) tended to contain tens to thousands of SARS-CoV-2 virions (Figure 5B, Figure 5—figure supplement 1E).

**Figure 5—figure supplement 1.**
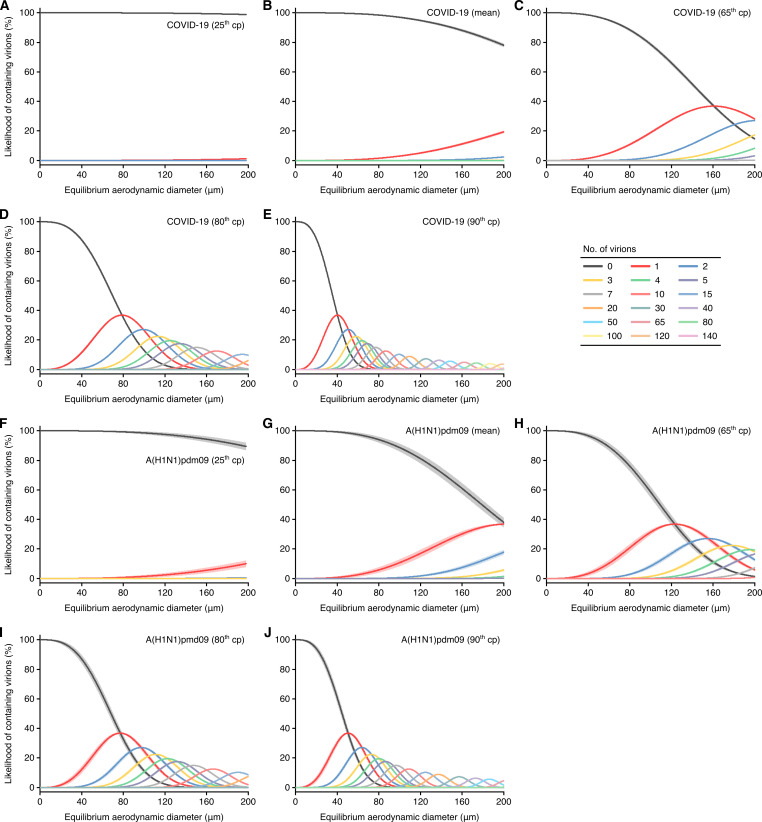
Likelihood of respiratory particles containing SARS-CoV-2 or A(H1N1)pdm09. (**A–E**) Estimated likelihood that droplets and aerosols contain viable SARS-CoV-2 when expelled by the 25th case percentile (cp) (**A**), mean (**B**), 65th cp (**C**), 80th cp (**D**) or 90th cp (**E**) for COVID-19 cases during the infectious period. (**F–J**) Estimated likelihood that droplets and aerosols contain viable A(H1N1)pdm09 when expelled by the 25th cp (**F**), mean (**G**), 65th cp (**H**), 80th cp (**I**) or 90th cp (**J**) for A(H1N1)pmd09 cases during the infectious period. For higher number of virions, some likelihood curves were omitted to aid visualization. Equilibrium particle diameters were taken to be 0.3 times the hydrated diameter during atomization. When the likelihood for zero virions approaches 0%, particles are expected to contain at least one viable copy. Lines and bands represent estimates and 95% confidence intervals, respectively, for estimated likelihoods.

**Figure 5—figure supplement 2.**
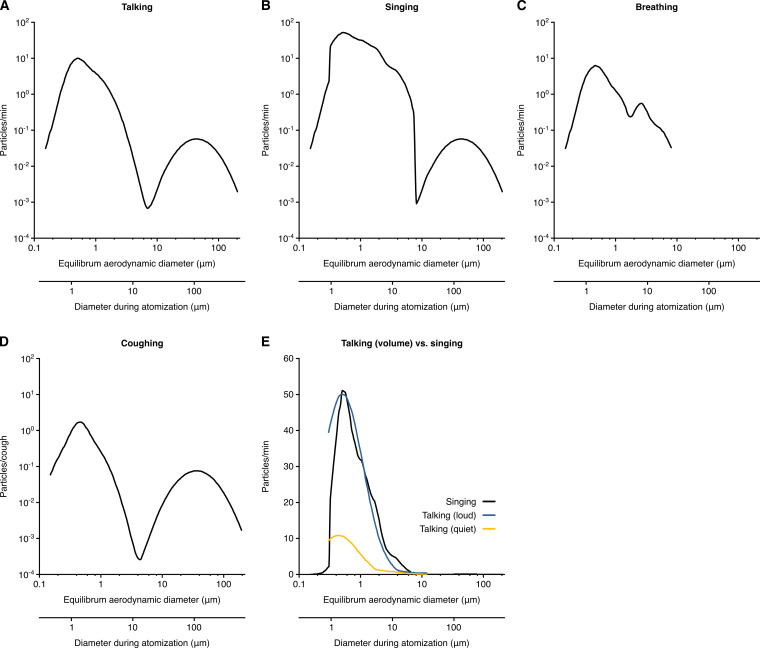
Rate profiles for particle expelled by respiratory activities. (**A–D**) Rate profiles of particles expelled while talking (**A**), singing (**B**), breathing (**C**) and coughing (**D**). (**E**) Comparison of the rate profiles of aerosol emission from singing and different amplitudes of talking. The rate profiles were calculated from the normalized concentrations in Johnson et al., 2011 (**A, B, D and E**) and Morawska et al., 2009 (**C**) or collected from Asadi et al. (2019) (**E**). Equilibrium particle diameters were taken to be 0.3 times the hydrated diameter during atomization. Breathing was taken to expel negligible quantities of larger particles based on the bronchiolar fluid film burst mechanism.

**Figure 5—figure supplement 3.**
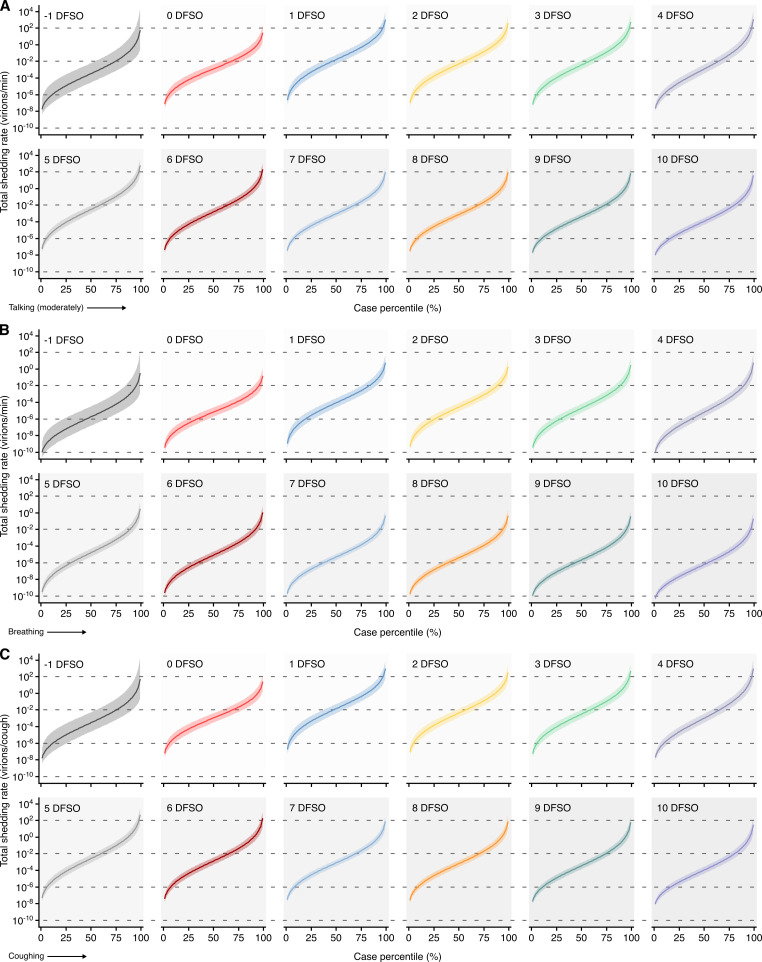
Heterogeneity in shedding SAR-CoV-2 via talking, breathing and coughing. (**A–C**) Case heterogeneity in the total SARS-CoV-2 shedding rate (over all particle sizes) by talking at a moderate amplitude (**A**), breathing (**B**) or coughing (**C**) for COVID-19 cases across the infectious period. Earlier presymptomatic days were excluded based on low specimen numbers. Data represent estimated rates for viable virus and range between the 1st and 99thcase percentiles. Lines and bands represent estimates and 95% confidence intervals, respectively.

**Figure 5—figure supplement 4.**
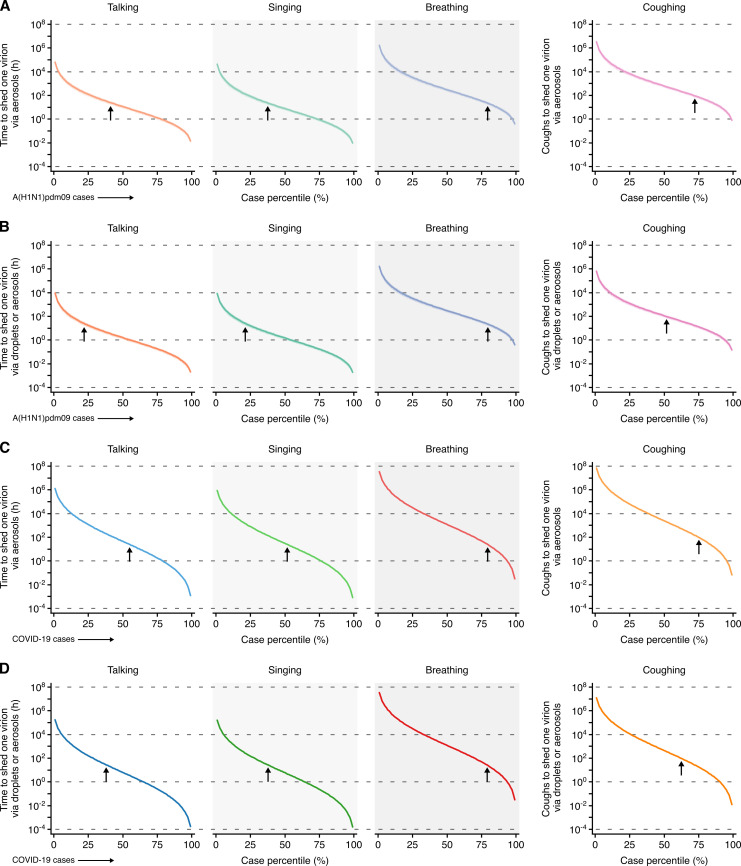
Heterogeneity in infectiousness for COVID-19 and A(H1N1)pmd09 cases during the infectious period. (**A, B**) Estimated time for a A(H1N1)pdm09 case to expel one virion via only aerosols (**A**) or either droplets or aerosols (**B**) by talking, singing, breathing or coughing. (**C, D**) Estimated time for a COVID-19 case to expel one SARS-CoV-2 virion via only aerosols (**C**) or either droplets or aerosols (**D**) by talking, singing, breathing or coughing. Data represent estimated times to expel viable virus and range between the 1st and 99th case percentiles (cps). Vertical arrows depict the cp expected to shed one virion in 24 hr (talking, singing or breathing) or 100 coughs. Lines and bands represent estimates and 95% confidence intervals, respectively.

**Figure 5—figure supplement 5.**
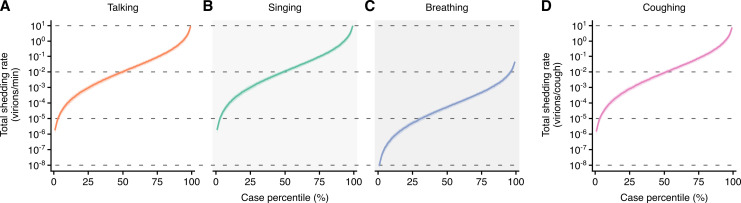
Heterogeneity in shedding A(H1N1)pdm09 via droplets and aerosols. (**A–D**) Case heterogeneity in the total A(H1N1)pdm09 shedding rates while talking (**A**), singing (**B**), breathing (**C**) and coughing (**D**) during the infectious period. Data represent estimated rates for viable virus and range between the 1st and 99thcase percentiles. Lines and bands represent estimates and 95% confidence intervals, respectively.

### Shedding SARS-CoV-2 via respiratory droplets and aerosols

Using the partitioning estimates in conjunction with published profiles of the particles expelled by respiratory activities (Figure 5—figure supplement 2), we next modeled the rates at which talking, singing, breathing and coughing shed viable SARS-CoV-2 across *d*_a_ (Figure 5C-F). Singing shed virions more rapidly than talking based on the increased emission of aerosols. Voice amplitude, however, had a significant effect on aerosol production, and talking loudly emitted aerosols at similar rates to singing (Figure 5—figure supplement 2E). Based on the generation of larger aerosols and droplets, talking and singing shed virions significantly more rapidly than breathing (Figure 5C-E). Each cough shed similar quantities of virions as in a minute of talking (Figure 5C, F).

Each of these respiratory activities expelled aerosols at greater rates than droplets, but particle size correlated with the likelihood of containing virions according to our model. Talking, singing and coughing expelled virions at comparable proportions via droplets (55.6–59.4%) and aerosols (40.6–44.4%), whereas breathing did so predominantly within aerosols (Figure 5G). Moreover, short-range aerosols mediated most of the virions (79.2–81.9%) shed via aerosols while talking normally and coughing. In comparison, while singing, or talking loudly, buoyant (14.5%) and long-range (17.5%) aerosols carried a larger proportion of the virions shed via aerosols (Figure 5G).

### Influence of heterogeneity in rVL on individual infectiousness

To interpret how heterogeneity in rVL influences individual infectiousness, we modeled total SARS-CoV-2 shedding rates (over all particle sizes) for each respiratory activity (Figure 5H, Figure 5—figure supplement 3). Between the 1st and the 99th cps, the estimates for a respiratory activity spanned ≥8.48 orders of magnitude on each DFSO; cumulatively from −1 to 10 DFSO, they spanned 11.0 orders of magnitude. Hence, many COVID-19 cases inherently presented minimal transmission risk, whereas highly infectious individuals shed considerable quantities of SARS-CoV-2. For the 98th cp at 1 DFSO, singing expelled 313 (95% CI: 37.5–3158) virions/min to the ambient environment, talking emitted 293 (35.1–2664) virions/min, breathing exhaled 1.54 (0.18–15.5) virions/min and coughing discharged 249 (29.8–25111) virions/cough; these estimates were approximately two orders of magnitude greater than those for the 85th cp. For the 98th cp at −1 DFSO, singing shed 14.5 (0.15–4515) virions/min and breathing exhaled 7.13 × 10^−2^ (7.20 × 10^−4^–220.2) virions/min. The estimates at 9–10 DFSO were similar to these presymptomatic ones (Figure 5H, Figure 5—figure supplement 3B). As indicated by comparable mean rVLs (Figure 3) and heterogeneities in rVL (Figure 4B, C), adult, pediatric, symptomatic/presymptomatic and asymptomatic COVID-19 subgroups presented similar distributions for shedding virions through these activities.

We also compared the influence of case variation on individual infectiousness between A(H1N1)pdm09 and COVID-19. Aerosol spread accounted for approximately half of A(H1N1)pdm09 transmission events (Cowling et al., 2013), and the 50% human infectious dose for aerosolized influenza A virus is approximately 1–3 virions in the absence of neutralizing antibodies (Fabian et al., 2008). Based on the model, 62.9% of A(H1N1)pdm09 cases were infectious (shed ≥1 virion) via aerosols within 24 hr of talking loudly or singing (Figure 5—figure supplement 4A), and the estimate was 58.6% within 24 hr of talking normally and 22.3% within 24 hr of breathing. In comparison, 48.0% of COVID-19 cases shed ≥1 virion via aerosols in 24 hr of talking loudly or singing (Figure 5—figure supplement 4C). Notably, only 61.4% of COVID-19 cases shed ≥1 virion via either droplets or aerosols in 24 hr of talking loudly or singing (Figure 5—figure supplement 4D). While the human infectious dose of SARS-CoV-2 by any exposure route remains unelucidated, it must be at least one viable copy. Thus, at least 38.6% of COVID-19 cases were expected to present negligible risk to spread SARS-CoV-2 through either droplets or aerosols in 24 hr. The proportion of potentially infectious cases further decreased as the threshold increased: 55.8, 42.5 and 25.0% of COVID-19 cases were expected to shed ≥2, ≥10 and ≥100 virions, respectively, in 24 hr of talking loudly or singing during the infectious period.

While these analyses indicated that a greater proportion of A(H1N1)pdm09 cases were inherently infectious, 18.8% of COVID-19 cases shed virions more rapidly than those infected with A(H1N1)pdm09 (Figure 4A). At the 98th cp for A(H1N1)pdm09, singing expelled 4.38 (2.85–6.78) virions/min and breathing exhaled 2.15 × 10^−2^ (1.40 × 10^−2^–30.34×10^−2^) virions/min. Highly infectious COVID-19 cases expelled virions at rates that were up to 1–2 orders of magnitude greater than their A(H1N1)pdm09 counterparts (Figure 5H, Figure 5—figure supplement 5).

## Discussion

This study provided systematic analyses of several factors characterizing SARS-CoV-2 transmissibility. First, our results indicate that broader heterogeneity in rVL facilitates greater overdispersion for SARS-CoV-2 than A(H1N1)pdm09. They suggest that many COVID-19 cases infect no one (Bi et al., 2020; Endo et al., 2020; Laxminarayan et al., 2020) because they inherently present minimal transmission risk via respiratory droplets or aerosols, although behavioral and environmental factors may further influence risk. Meanwhile, highly infectious cases can shed tens to thousands of SARS-CoV-2 virions/min, especially between 1 and 5 DFSO, potentiating superspreading events. The model estimates, when corrected to copies rather than virions, align with recent clinical findings for exhalation rates of SARS-CoV-2 (Ma et al., 2020). In comparison, a greater proportion of A(H1N1)pdm09 cases are infectious but shed virions at low rates, which concurs with more uniform transmission and few superspreading events observed during the 2009 H1N1 pandemic (Brugger and Althaus, 2020; Roberts and Nishiura, 2011). Moreover, our analyses suggest that heterogeneity in rVL may be generally associated with overdispersion for viral respiratory infections. In this case, rVL distribution can serve as an early correlate for transmission patterns, including superspreading, during outbreaks of novel respiratory viruses. When considered jointly with contact-tracing studies, this provides epidemiological triangulation on *k*: heterogeneity in rVL indirectly estimates *k* via an association, whereas contact tracing empirically characterizes transmission chains to estimate *k* but is limited by incomplete or incorrect recall of contact events by cases. When transmission is highly overdispersed, targeted interventions may disproportionately mitigate infection (Lee et al., 2020), with models showing that focused control efforts on the most infectious cases outperform random control policies (Lloyd-Smith et al., 2005).

Second, we analyzed SARS-CoV-2 kinetics during respiratory infection. While heterogeneity remains broad throughout the infectious period, rVL tends to peak at 1 DFSO and be elevated for 1–5 DFSO, coinciding with the period of highest attack rates observed among close contacts (Cheng et al., 2020). These results indicate that transmission risk tends to be greatest near, and soon after, illness rather than in the presymptomatic period, which concurs with large tracing studies (6.4–12.6% of secondary infections from presymptomatic transmission) (Du et al., 2020; Wei et al., 2020) rather than early temporal models (~44%) (He et al., 2020). Furthermore, our kinetic analysis suggests that, on average, SARS-CoV-2 reaches diagnostic concentrations 1.54–3.17 days after respiratory infection (−3.84 to −2.21 DFSO), assuming assay detection limits of 1–3 log_10_ copies/ml, respectively, for nasopharyngeal swabs immersed in 1 ml of transport media.

Third, we assessed the relative infectiousness of COVID-19 subgroups. As a common symptom of COVID-19 (Guan et al., 2020), coughing sheds considerable numbers of virions via droplets and short-range aerosols. Thus, symptomatic infections tend to be more contagious than asymptomatic ones, providing one reason as to why asymptomatic cases transmit SARS-CoV-2 at lower relative rates (Li et al., 2020b), especially in close contact (Luo et al., 2020), despite similar rVLs and increased contact patterns. Accordingly, children (48–54% of symptomatic cases present with cough) (Lu et al., 2020b; Team and CDC COVID-19 Response Team, 2020) may be less contagious than adults (68–80%) (Guan et al., 2020; Team and CDC COVID-19 Response Team, 2020) based on tendencies of symptomatology rather than rVL. Conversely, coughing sheds few virions via smaller aerosols. While singing and talking loudly, highly infectious cases can shed tens to hundreds of SARS-CoV-2 virions/min via long-range and buoyant aerosols.

Our study has limitations. The systematic search found a limited number of studies reporting quantitative specimen measurements from the presymptomatic period, meaning that these estimates may be sensitive to sampling bias. Although additional studies have reported semiquantitative metrics (cycle thresholds), these data were excluded because they cannot be compared on an absolute scale due to batch effects (Han et al., 2021), limiting use in compound analyses. In addition, our models considered virion partitioning during atomization to be a Poisson process, which stochastically associates partitioning with particle volume. Partitioning mechanisms associated with surface area, perhaps such as film bursting (Bird et al., 2010; Johnson and Morawska, 2009), may enrich the quantities of virions in smaller aerosols, based on their surface area-to-volume ratio. As severe COVID-19 is associated with high, persistent SARS-CoV-2 shedding in the lower respiratory tract (Chen et al., 2021) and small particles are typically generated there (Johnson et al., 2011), severe cases may also expel higher quantities of virions via smaller aerosols.

Furthermore, this study considered population-level estimates of the infectious periods, viability proportions and profiles for respiratory particles, which omit individual or environmental variation. Studies differ in their measurements of the emission rates and size distributions of the particles expelled during respiratory activities (Johnson et al., 2011; Schijven et al., 2020). Their characterization methods may prompt these differences, or they may be due to individual variation, including from distinctions in respiratory capacity, especially for young children, and phonetic tendencies (Asadi et al., 2020). Some patients shed SARS-CoV-2 with diminishing viability soon after symptom onset (Wölfel et al., 2020), whereas others produce replication-competent virus for weeks (van Kampen et al., 2021). The proportion of viable SARS-CoV-2 in respiratory particles, and how case characteristics or environmental factors influence it, remains under investigation (Fears et al., 2020; Lednicky et al., 2020; Morris et al., 2020). Cumulatively, these sources of variation may influence the shedding model estimates, further increasing heterogeneity in individual infectiousness.

Taken together, our findings provide a potential path forward for disease control. While talking, singing and coughing, our models indicate that SARS-CoV-2 is shed via droplets (55.6–59.4% of shed virions), short-range aerosols (30.1–34.9%), long-range aerosols (7.7–8.3%) and buoyant aerosols (0.01–6.5%). Transmission, however, requires exposure. For direct transmission, droplets tend to be sprayed ballistically onto susceptible tissue. Aerosols can be inhaled, may penetrate more deeply into the lungs and more easily facilitate superspreading events. However, with short durations of stay in well-ventilated areas, the exposure risk for both droplets and aerosols remains correlated with proximity to infectious cases (Liu et al., 2017a; Prather et al., 2020). Strategies to abate infection should limit crowd numbers and duration of stay while reinforcing distancing, low-voice amplitudes and widespread mask usage; well-ventilated settings can be recognized as lower-risk venues. Coughing can shed considerable quantities of virions, while rVL tends to peak at 1 DFSO and can be high throughout the infectious period. Thus, immediate, sustained self-isolation upon illness is crucial to curb transmission from symptomatic cases. Collectively, our analyses highlight the role of cases with high rVLs in propelling the COVID-19 pandemic. While diagnosing COVID-19, qRT-PCR can also triage contact tracing, prioritizing these patients: for nasopharyngeal swabs immersed in 1 ml of transport media, ≥7.14 (95% CI: 7.07–7.22) log_10_ copies/ml corresponds to the top 20% of COVID-19 cases for variants before August 2020. Doing so may identify asymptomatic and presymptomatic infections more efficiently, a key step towards mitigation and elimination as the pandemic continues.

## Materials and methods

### Search strategy, selection criteria and data collection

We undertook a systematic review and prospectively submitted the protocol for registration on PROSPERO (registration number, CRD42020204637). Other than the title of this study, we have followed PRISMA reporting guidelines (Moher et al., 2009). The systematic review was conducted according to Cochrane methods guidance (Higgins et al., 2019).

The search included papers that (i) reported positive, quantitative measurements (copies/ml or an equivalent metric) of SARS-CoV-2, SARS-CoV-1 or A(H1N1)pdm09 in human respiratory specimens (endotracheal aspirate [ETA], nasopharyngeal aspirate [NPA], nasopharyngeal swab [NPS], oropharyngeal swab [OPS], posterior oropharyngeal saliva [POS] and sputum [Spu]) from COVID-19, SARS or A(H1N1)pdm09 cases; (ii) reported data that could be extracted from the estimated infectious periods of SARS-CoV-2 (defined as −3 to +10 DFSO for symptomatic cases and 0 to +10 days from the day of laboratory diagnosis for asymptomatic cases), SARS-CoV-1 (defined as 0 to +20 DFSO or the equivalent asymptomatic period) or A(H1N1)pdm09 (defined as −2 to +9 DFSO for symptomatic cases and 0 days to +9 days from the day of laboratory diagnosis for asymptomatic cases); and (iii) reported data for two or more cases with laboratory-confirmed COVID-19, SARS or A(H1N1)pdm09 based on World Health Organization (WHO) case definitions. Quantitative specimen measurements were considered after RNA extraction for diagnostic sequences of SARS-CoV-2 (*Ofr1b*, *N*, *RdRp* and *E* genes), SARS-CoV-1 (*Ofr1b*, *N* and *RdRp* genes) and A(H1N1)pdm09 (*HA* and *M* genes).

Studies were excluded, in the following order, if they (i) studied an ineligible disease; (ii) had an ineligible study design, including those that were reviews of evidence (e.g., scoping, systematic or narrative), did not include primary clinical human data, reported data for less than two cases due to an increased risk of selection bias, were incomplete (e.g., ongoing clinical trials), did not report an RNA extraction step before measurement or were studies of environmental samples; (iii) reported an ineligible metric for specimen concentration (e.g., qualitative RT-PCR or cycle threshold [Ct] values without calibration included in the study); (iv) reported quantitative measurements from an ineligible specimen type (e.g., blood specimens, pooled specimens or self-collected POS or Spu patient specimens in the absence of a healthcare professional); (v) reported an ineligible sampling period (consisted entirely of data that could not be extracted from within the infectious period); or (vi) were duplicates of an included study (e.g., preprinted version of a published paper or duplicates not identified by Covidence). We included data from control groups receiving standard of care in interventional studies but excluded data from the intervention group. Patients in the intervention group are, by definition, systematically different from general case populations because they receive therapies not being widely used for treatment, which may influence virus concentrations. Interventional studies examining the comparative effectiveness of two or more treatments were excluded for the same reason. Studies exclusively reporting semiquantitative measurements (e.g., Ct values) of specimen concentration were excluded as these measurements are sensitive to batch and instrument variation and, without proper calibration, cannot be compared on an absolute scale across studies (Han et al., 2021).

We searched, without the use of filters or language restrictions, the following sources: MEDLINE (via Ovid, 1946 to 7 August 2020), EMBASE (via Ovid, 1974 to 7 August 2020), Cochrane Central Register of Controlled Trials (via Ovid, 1991 to 7 August 2020), Web of Science Core Collection (including Science Citation Index Expanded, 1900 to 7 August 2020; Social Sciences Citation Index, 1900 to 7 August 2020; Arts & Humanities Citation Index, 1975 to 7 August 2020; Conference Proceedings Citation Index – Science, 1990 to 7 August 2020; Conference Proceedings Citation Index – Social Sciences & Humanities, 1990 to 7 August 2020; and Emerging Sources Citation Index, 2015 to 7 August 2020), as well as medRxiv and bioRxiv (both searched through Google Scholar via the Publish or Perish program, to 7 August 2020). We also gathered studies by searching through the reference lists of review articles identified by the database search, by searching through the reference lists of included articles, through expert recommendation (by Eric J. Topol and Akiko Iwasaki on Twitter) and by hand-searching through journals (*Nature*, *Nat. Med.*, *Science*, *NEJM*, *Lancet*, *Lancet Infect. Dis.*, *JAMA*, *JAMA Intern. Med.* and *BMJ*). A comprehensive search was developed by a librarian, which included subject headings and keywords. The search strategy had three main concepts (disease, specimen type and outcome), and each concept was combined using the appropriate Boolean operators. The search was tested against a sample set of known articles that were pre-identified. The line-by-line search strategies for all databases are included in Figure 1—source data 1, Figure 1—source data 2, Figure 1—source data 3, Figure 1—source data 4, Figure 1—source data 5. The search results were exported from each database and uploaded to the Covidence online system for deduplication and screening.

Two authors independently screened titles and abstracts, reviewed full texts, collected data and assessed risk of bias via Covidence and a hybrid critical appraisal checklist based on the Joanna Briggs Institute (JBI) tools for case series, analytical cross-sectional studies and prevalence studies (Moola et al., 2020; Munn et al., 2019; Munn et al., 2015). To evaluate the sample size in a study, we used the following calculation:

(1) $$n^{*}=\frac{z^{2}\sigma }{d^{2}},$$

where $n^{*}$ is the sample size threshold, z is the z-score for the level of confidence (95%), σ is the standard deviation (assumed to be 3 log_10_ copies/ml, one quarter of the full range of rVLs) and d is the marginal error (assumed to be 1 log_10_ copies/ml, based on the minimum detection limit for qRT-PCR across studies) (Johnston et al., 2019). The hybrid JBI critical appraisal checklist is shown in the Appendix. Studies were considered to have low risk of bias if they met the majority of the items, indicating that the estimates were likely to be correct for the target population. Inconsistencies were resolved by discussion and consensus.

The search found 29 studies for COVID-19 (Argyropoulos et al., 2020; Baggio et al., 2020; Fajnzylber et al., 2020; Han et al., 2020a; Han et al., 2020b; Hung et al., 2020; Hurst et al., 2020; Iwasaki et al., 2020; Kawasuji et al., 2020; L'Huillier et al., 2020; Lavezzo et al., 2020; Lennon et al., 2020; Lucas et al., 2020; Mitjà et al., 2020; Pan et al., 2020; Peng et al., 2020; Perera et al., 2020; Shi et al., 2020; Shrestha et al., 2020; To et al., 2020; van Kampen et al., 2021; Vetter et al., 2020; Wölfel et al., 2020; Wyllie et al., 2020; Xu et al., 2020; Yonker et al., 2020; Zhang et al., 2020a; Zheng et al., 2020; Zou et al., 2020), 8 studies for SARS (Chen et al., 2006; Cheng et al., 2004; Chu et al., 2005; Chu et al., 2004; Hung et al., 2004; Peiris et al., 2003; Poon et al., 2004; Poon et al., 2003) and 27 studies for A(H1N1)pdm09 (Chan et al., 2011; Cheng et al., 2010; Cowling et al., 2010; Duchamp et al., 2010; Esposito et al., 2011; Hung et al., 2010; Ip et al., 2016; Ito et al., 2012; Killingley et al., 2010; Launes et al., 2012; Lee et al., 2011a; Lee et al., 2011b; Li et al., 2010a; Li et al., 2010b; Loeb et al., 2012; Lu et al., 2012; Meschi et al., 2011; Ngaosuwankul et al., 2010; Rath et al., 2012; Rodrigues Guimarães Alves et al., 2020; Suess et al., 2010; Thai et al., 2014; To et al., 2010a; To et al., 2010b; Watanabe et al., 2011; Wu et al., 2012; Yang et al., 2011), and data were collected from each study. For preprinted studies that were published as journal articles before the revised submission of this manuscript, we included the citation for the journal article. Descriptive statistics on quantitative specimen measurements were collected from confirmed cases directly if reported numerically or using WebPlotDigitizer 4.3 (https://apps.automeris.io/wpd/) if reported graphically. Individual specimen measurements were collected directly if reported numerically or, when the data were clearly represented, using the tool if reported graphically. We also collected the relevant numbers of cases, types of cases, reported treatments, volumes of transport media, numbers of specimens and DFSO (for symptomatic cases) or day relative to initial laboratory diagnosis (for asymptomatic cases) on which each specimen was taken. Hospitalized cases were defined as those being tested in a hospital setting and then admitted. Non-admitted cases were defined as those being tested in a hospital setting but not admitted. Community cases were defined as those being tested in a community setting. Symptomatic, presymptomatic and asymptomatic infections were defined as in the study. Based on rare description in contributing studies, paucisymptomatic infections, when described, were included with symptomatic ones. Pediatric cases were defined as those below 18 years of age or as defined in the study. Adult cases were defined as those 18 years of age or higher or as defined in the study.

### Calculation of rVLs from specimen measurements

In this study, viral concentrations in respiratory specimens were denoted as specimen measurements, whereas viral concentrations in the respiratory tract were denoted as rVLs. To determine rVLs, each collected quantitative specimen measurement was converted to rVL based on the dilution factor. For example, measurements from swabbed specimens (NPS and OPS) typically report the RNA concentration in viral transport media. Based on the expected uptake volume for swabs (0.128 ± 0.031 ml, mean ± SD) (Warnke et al., 2014) or reported collection volume for expulsed fluid in the study (e.g., 0.5–1 ml) along with the reported volume of transport media in the study (e.g., 1 ml), we calculated the dilution factor for each respiratory specimen to estimate the rVL. If the diluent volume was not reported, then the dilution factor was calculated assuming a volume of 1 ml (NPS and OPS), 2 ml (POS and ETA) or 3 ml (NPA) of transport media (Lavezzo et al., 2020; Poon et al., 2004; To et al., 2020). Unless dilution was reported for Spu specimens, we used the specimen measurement as the rVL (Wölfel et al., 2020). The non-reporting of diluent volume was noted as an element increasing risk of bias in the hybrid JBI critical appraisal checklist. Specimen measurements (based on instrumentation, calibration, procedures and reagents) are not standardized and, as DFSO is typically based on patient recall, there is also inherent uncertainty in these values. While the above procedures (including only quantitative measurements after extraction as an inclusion criterion, considering assay detection limits and correcting for specimen dilution) have considered many of these factors, non-standardization remains an inherent limitation in the variability of specimen measurements.

### Meta-regression of *k* and heterogeneity in rVL

To assess the relationship between *k* and heterogeneity in rVL, we performed a univariate meta-regression (log⁡k=a*SD+b, where a is the slope for association and b is the intercept) between pooled estimates of *k* (based on studies describing community transmission) for COVID-19 (*k* = 0.409) (Adam et al., 2020; Tariq et al., 2020; Zhang et al., 2020b; Laxminarayan et al., 2020; Bi et al., 2020; Endo et al., 2020; Riou and Althaus, 2020), SARS (*k* = 0.165) (Lloyd-Smith et al., 2005) and A(H1N1)pdm09 (*k* = 8.155) (Brugger and Althaus, 2020; Roberts and Nishiura, 2011) and the SD of the rVLs in contributing studies. Since SD was the metric, we used a fixed-effects model. For weighting in the meta-regression, we used the proportion of rVL samples from each study relative to the entire systematic dataset ($W_{i}=n_{i}/n_{total}$). All calculations were performed in units of log_10_ copies/ml. As the meta-regression used pooled estimates of *k* for each infection, it assumed that there was no correlated bias to *k* across contributing studies. The limit of detection for qRT-PCR instruments used in the included studies did not significantly affect the analysis of heterogeneity in rVL as these limits tended to be below the values found for specimens with low virus concentrations. The meta-regression was conducted using all contributing studies and showed a weak association. Meta-regression was also conducted using studies that had low risk of bias according to the hybrid JBI critical appraisal checklist and showed a strong association. The p-value for association was obtained using the meta-regression slope *t*-test for a, the effect estimate. While there is intrinsic measurement error in virus quantitation, based on the systematic review protocol and study design (as described above), this error should similarly increase heterogeneity in rVL for each virus, and the difference in heterogeneity in rVL between viruses should arise from the viruses.

### Meta-analysis of rVLs

Based on the search design and composition of contributing studies, the meta-analysis overall estimates were the expected SARS-CoV-2, SARS-CoV-1 and A(H1N1)pdm09 rVL when encountering a COVID-19, SARS or A(H1N1)pdm09 case, respectively, during their infectious period. Pooled estimates and 95% CIs for the expected rVL of each virus across their infectious period were calculated using a random-effects meta-analysis (DerSimonian and Laird method). For studies reporting summary statistics in medians and interquartile or total ranges, we derived estimates of the mean and variance and calculated the 95% CIs (Wan et al., 2014). All calculations were performed in units of log_10_ copies/ml. Between-study heterogeneity in meta-analysis was assessed using Cochran’s *Q* test and the *I*^2^ and τ^2^ statistics. If significant between-study heterogeneity in meta-analysis was encountered, sensitivity analysis based on the risk of bias of contributing studies was performed. The meta-analyses were conducted using STATA 14.2 (StataCorp LLC, College Station, TX, USA).

### Age and symptomatology subgroup analyses of SARS-CoV-2 rVLs

The overall estimate for each subgroup was the expected rVL when encountering a case of that subgroup during the infectious period. Studies reporting data exclusively from a subgroup of interest were directly included in the analysis after rVL estimations. For studies in which data for these subgroups constituted only part of its dataset, rVLs from the subgroup were extracted to calculate the mean, variance and 95% CIs. Random-effects meta-analysis was performed as described above. For meta-analyses of pediatric and asymptomatic COVID-19 cases, contributing studies had low risk of bias, and no risk-of-bias sensitivity analyses were performed for these subgroups.

### Distributions of rVL

We pooled the entirety of individual sample data in the systematic dataset by disease, COVID-19 subgroups and DFSO. For analyses of SARS-CoV-2 dynamics across disease course, we included estimated rVLs from negative qRT-PCR measurements of respiratory specimens for cases that had previously been quantitatively confirmed to have COVID-19. These rVLs were estimated based on the reported assay detection limit in the respective study. Probability plots and modified Kolmogorov–Smirnov tests used the Blom scoring method and were used to determine the suitability of normal, lognormal, gamma and Weibull distributions to describe the distribution of rVLs for SARS-CoV-2, SARS-CoV-1 and A(H1N1)pdm09. For each virus, the data best conformed to Weibull distributions, which is described by the probability density function

(2) $$f\left(\upsilon \right)=\frac{\alpha }{\beta }{\left(\frac{\upsilon }{\beta }\right)}^{\alpha -1}e^{-{\left(\upsilon /\beta \right)}^{\alpha }},$$

where α is the shape factor, β is the scale factor and υ is rVL (υ≥0 log_10_ copies/ml). Weibull distributions were fitted on the entirety of collected individual sample data for the respective category. Since individual specimen measurements could not be collected from all studies, there was a small bias on the mean estimate for each fitted distribution. Thus, for the curves shown in Figure 4B, C, the mean of the Weibull distributions summarized in Figure 4—figure supplement 2 was adjusted to be the subgroup meta-analysis estimate for correction; the SD and distribution around that mean remained consistent.

For each Weibull distribution, the value of the rVL at the x th percentile was determined using the quantile function,

(3) $$\upsilon_{x}=\beta {\left[-\ln\left(1-x\right)\right]}^{1/\alpha }.$$

For cp curves, we used Equation (3) to determine rVLs from the 1st cp to the 99th cp (step size, 1%). Curve fitting to Equation (2) and calculation of Equation (3) and its 95% CI was performed using the Distribution Fitter application in Matlab R2019b (MathWorks, Inc, Natick, MA, USA).

### Viral kinetics

To model SARS-CoV-2 kinetics during respiratory infection, we used a mechanistic epithelial cell-limited model for the respiratory tract (Baccam et al., 2006), based on the system of differential equations:

(4) $$\frac{dT}{dt}=-\beta TV$$

(5) $$\frac{dI}{dt}=\beta TV-\delta I$$

(6) $$\frac{dV}{dt}=pI-cV,$$

where T is the number of uninfected target cells, I is the number of productively infected cells, V is the rVL, β is the infection rate constant, p is the rate at which airway epithelial cells shed virus to the extracellular fluid, c is the clearance rate of virus and δ is the clearance rate of productively infected cells. Using these parameters, the viral half-life in the respiratory tract ($t_{1/2}=\ln2/c$) and the half-life of productively infected cells ($t_{1/2}=\ln2/\delta$) could be estimated. Moreover, the cellular basic reproductive number (the expected number of secondary infected cells from a single productively infected cell placed in a population of susceptible cells) was calculated by

(7) $$R_{0,c}=\frac{p\beta T_{0}}{c\delta },$$

For initial parameterization, Equations (4)–(6) were simplified according to a quasi-steady state approximation (Ikeda et al., 2016) to

(8) $$\frac{dT}{dt}=-\beta TV$$

(9) $$\frac{dV}{dt}=rTV-\delta V,$$

where r=pβ/c, for a form with greater numerical stability. The system of differential equations was fitted on the mean estimates of SARS-CoV-2 rVL between -2 and 10 DFSO using the entirety of individual sample data in units of copies/ml. Numerical analysis was implemented using the Fit ODE app in OriginPro 2019b (OriginLab Corporation, Northampton, MA, USA) via the Runge–Kutta method and initial parameters $V_{0}$, $I_{0}$ and $T_{0}$ of 4 copies/ml, 0 cells and 5 × 10^7^ cells, respectively, for the range –5 to 10 DFSO. The analysis was first performed with Equations (8) and (9). These output parameters were then used to initialize final analysis using Equations (4)–(6), where the estimates for β and δ were input as fixed and variable parameters, respectively. The fitted line and its coefficient of determination (*r*^2^) were presented. The estimated half-life of SARS-CoV-2 RNA has a skewed 95% CI (Figure 4—figure supplement 4). As c is in the denominator of the equation for half-life ($t_{1/2}=\ln2/c$), $t_{1/2}$ is sensitive to *c* below 1, which is the case for its lower 95% CI (Figure 4—figure supplement 4) and the source of the skew.

To estimate the average incubation period, we extrapolated the kinetic model to 0 log_10_ copies/ml pre-symptom onset. To estimate the average duration of shedding, we extrapolated the model to 0 log_10_ copies/ml post-symptom onset. Unlike in experimental studies, this estimate for duration of shedding was not defined by assay detection limits. To estimate the average DFSO on which SARS-CoV-2 concentration reached diagnostic levels, we extrapolated the model pre-symptom onset to the equivalent of 1 and 3 log_10_ copies/ml (chosen as example assay detection limits) in specimen concentration for NPSs immersed in 1 ml of transport media, as described by the dilution factor estimation above. The average time from respiratory infection to reach diagnostic levels was then calculated by subtracting these values from the estimated average incubation period. The extrapolated time for SARS-CoV-2 to reach diagnostic concentrations in the respiratory tract should be validated in tracing studies, in which contacts are prospectively subjected to daily sampling.

### Likelihood of respiratory particles containing virions

To calculate an unbiased estimator for viral partitioning (the expected number of viable copies in an expelled particle at a given size), we multiplied rVLs with the volume equation for spherical particles during atomization and the estimated viability proportion, according to the following equation:

(10) $$\lambda =\frac{\pi \rho v_{p}\gamma \upsilon }{6}d^{3},$$

where λ is the expectation value, ρ is the material density of the respiratory particle (997 kg/m^3^), $v_{p}$ is the volumetric conversion factor (1 ml/g), γ is the viability proportion, υ is the rVL and d is the hydrated diameter of the particle during atomization.

The model assumed γ was 0.1% as a population-level estimate. For influenza, approximately 0.1% of copies in particles expelled from the respiratory tract represent viable virus (Yan et al., 2018), which is equivalent to one viable copy in 3 log_10_ copies/ml for rVL or, after dilution in transport media, roughly one in 4 log_10_ copies/ml for specimen concentration. Respiratory specimens taken from influenza cases show positive cultures for specimen concentrations down to 4 log_10_ copies/ml (Lau et al., 2010). Likewise, for COVID-19 cases, recent reports also show culture-positive respiratory specimens with SARS-CoV-2 concentrations down to 4 log_10_ copies/ml (Wölfel et al., 2020), including from pediatric (L'Huillier et al., 2020) and asymptomatic (Arons et al., 2020) cases. Moreover, replication-competent SARS-CoV-2 has been found in respiratory specimens taken throughout the respiratory tract (mouth, nasopharynx, oropharynx and lower respiratory tract) (Jeong et al., 2020; Wölfel et al., 2020). Taken together, these considerations suggested that the assumption for viability proportion (0.1%) was suitable to model the likelihood of respiratory particles containing viable SARS-CoV-2. In accordance with the discussion above, the model did not differentiate this population-level viability estimate based on age, symptomatology or sites of atomization. Based on the relative relationship between the residence time of expelled particles before assessment (~5 s) (Yan et al., 2018), we took the viability proportion to be for equilibrated particles.

Likelihood profiles were determined using Poisson statistics, as described by the probability mass function

(11) $$P\left(X=k\right)=\frac{\lambda^{k}e^{-\lambda }}{k!},$$

where k is the number of virions partitioned within the particle. For λ, 95% CIs were determined using the variance of its rVL estimate. To determine 95% CIs for likelihood profiles from the probability mass function, we used the delta method, which specifies

(12) $$Var\left(g\left(\theta \right)\right)\approx \sigma^{2}\overset{˙}{g}{\left(\theta \right)}^{\prime }D\overset{˙}{g}\left(\theta \right),$$

where $\sigma^{2}D$ is the covariance matrix of θ and $\dot{g}\left(\theta \right)$ is the gradient of $g\left(\theta \right)$. For the univariate Poisson distribution, $\sigma^{2}D=\lambda$ and

(13) $$\overset{˙}{g}\left(\theta \right)=\frac{\lambda^{k-1}e^{-\lambda }}{k!}\left(k-\lambda \right).$$

### Rate profiles of particles expelled by respiratory activities

Distributions from the literature were used to determine the rate profiles of particles expelled during respiratory activities. For breathing, talking and coughing, we used data from Johnson et al., 2011. For singing, we used data from Morawska et al., 2009 for smaller aerosols (*d*_a_ < 20 μm) and used the profiles from talking for larger aerosols and droplets based on the oral cavity mechanism from Johnson et al., 2011. Rate profiles (particles/min or particles/cough) were calculated based on the corrected normalized concentration (d*C*_n_/dlog*D*_p_, in units of particles/cm^3^) at each discrete particle size, normalization (32 size channels per decade) for the aerodynamic particle sizer used, unit conversion (cm^3^ to l) and the sample flow rate (1 l/min). For coughing, the calculation assumed that participants coughed 10 times in the 30-s sampling interval. To determine the corrected normalized concentrations for breathing, we used a particle dilution factor of 4 and evaporation factor of 0.5, consistent with the other respiratory activities in Johnson et al., 2011. Breathing was taken to expel negligible quantities of larger respiratory particles based on the bronchiolar fluid film burst mechanism (Johnson et al., 2011). To account for intermittent breathing while talking and singing, the rate profiles for these activities included the contribution of aerosols expelled by breathing. We compared these rate profiles with those collected from talking loudly and talking quietly from Asadi et al., 2020. In our models, we took the diameter of dehydrated respiratory particles to be 0.3 times the initial size when atomized in the respiratory tract (Johnson et al., 2011; Lieber et al., 2021; Liu et al., 2017b). Equilibrium aerodynamic diameter was calculated by $d_{a}=d_{p}{\left(\rho /\rho_{0}\right)}^{1/2}$, where $d_{p}$ is the dehydrated diameter, ρ is the material density of the respiratory particle and $\rho_{0}$ is the reference material density (1 g/cm^3^). Curves based on discrete particle measurements were connected using the nonparametric Akima spline function.

### Shedding virions via respiratory droplets and aerosols

To model the respiratory shedding rate across particle size, rVL estimates and the hydrated diameters of particles expelled by a respiratory activity were input into Equation (10), and the output was then multiplied by the rate profile of the activity (talking, singing, breathing or coughing). To assess the relative contribution of aerosols and droplets to mediating respiratory viral shedding for a given respiratory activity, we calculated the proportion of the cumulative hydrated volumetric rate contributed by buoyant aerosols (*d*_a_ ≤ 10 μm), long-range aerosols (10 μm < *d*_a_ ≤ 50 μm), short-range aerosols (50 μm < *d*_a_ ≤ 100 μm) and droplets (*d*_a_ > 10 μm) for that respiratory activity. Since the Poisson mean was proportional to cumulative volumetric rate, this estimate of the relative contribution of aerosols and droplets to respiratory viral shedding was consistent among viruses and cps in the model.

To determine the total respiratory shedding rate for a given respiratory activity across cp, we determined the cumulative hydrated volumetric rate (by summing the hydrated volumetric rates across particle sizes for that respiratory activity) of particle atomization and input it into Equation (10). Using rVLs and their variances as determined by the Weibull quantile functions, we then calculated the Poisson means and their 95% CIs at the different cps.

To assess the influence of heterogeneity in rVL on individual infectiousness, we first considered transmission of A(H1N1)pdm09 via aerosols (Cowling et al., 2013). The 50% human infectious dose (HID_50_) of aerosolized A(H1N1)pdm09 was taken to be 1–3 virions (Fabian et al., 2008). To determine the expected time required for a A(H1N1)pdm09 case to shed one virion via aerosols, we took the reciprocal of the Poisson means and their 95% CIs at the different cps of the estimated shedding rates. The expected time required for a COVID-19 case to shed one virion via aerosols or one virion via droplets or aerosols was determined in a same manner.

### Data availability

The systematic dataset and model outputs from this study were uploaded to Zenodo (https://zenodo.org/record/4658971). The code generated during this study is available at GitHub (https://github.com/paulzchen/sars2-heterogeneity; Chen, 2020; copy archived at swh:1:rev:06649ccfb6e92918b439332314ebf330abfa3d16). The systematic review protocol was prospectively registered on PROSPERO (registration number, CRD42020204637).

## Data Availability

The systematic dataset and model outputs from this study are uploaded to Zenodo (https://zenodo.org/record/4658971). The code generated during this study is available at GitHub (https://github.com/paulzchen/sars2-heterogeneity; copy archived at https://archive.softwareheritage.org/swh:1:rev:06649ccfb6e92918b439332314ebf330abfa3d16). The systematic review protocol was prospectively registered on PROSPERO (registration number, CRD42020204637). The following dataset was generated: ChenPZBobrovitzNPremjiZKoopmansMFismanDNGuFX2020Heterogeneity in transmissibility and shedding SARS-CoV-2 via droplets and aerosolsZenodo10.5281/zenodo/4658971PMC813983833861198

## Additional files

Transparent reporting form

## Appendix 1

**Appendix 1—table 1.** Characteristics of contributing studies.

Study*	Country	No. of cases included (no. of specimens)	No. of pediatric cases (no. of specimens)	No. of asymptomatic cases (no. of specimens)	Disease caused by virus (WHO case definition)	Treatments given (type)†	Individual data extracted (diluent volume reported)^‡^	Adjusted viral load^§^ (type of specimen)	Weight, % (meta-analysis category)^||^	Weight, % (meta-regression)	Risk of bias^¶^
Argyropoulos et al., 2020	USA	205 (205)	0	0	COVID-19 (confirmed)	N/A	No (no)	Yes (NPS)	3.80 (V), 5.09 (A), 3.98 (S/Ps)	2.13	********
Baggio et al., 2020	Switzerland	405 (405)	58 (58)	0	COVID-19 (confirmed)	N/A	Yes (no)	Yes (NPS)	3.84 (V), 5.14 (A), 13.9 (P), 3.88 (S/Ps)	4.20	*******
Fajnzylber et al., 2020	USA	- (31)	0	0	COVID-19 (confirmed)	Yes (remdesivir)	Yes (yes)	Yes (NPS, OPS) No (Spu)	3.45 (V), 4.62 (A), 3.76 (S/Ps)	0.32	********
Hung et al., 2020	South Korea	2 (8)	1 (6)	0	COVID-19 (confirmed)	N/A	Yes (no)	Yes (NPS, OPS)	2.53 (V), 4.06 (S/Ps)	0.08	******
Hung et al., 2020	South Korea	12 (27)	12 (27)	3 (7)	COVID-19 (confirmed)	N/A	Yes (no)	Yes (NPS)	3.43 (V), 17.5 (P), 4.10 (S/Ps), 14.3 (As)	0.28	********
Hung et al., 2020	China	41 (310)	0	0	COVID-19 (confirmed)	Yes (control group: lopinavir ritonavir, antimicrobial treatment for secondary bacterial infection as indicated clinically, hydrocortisone for those requiring oxygen support)	No (no)	Yes (NPS, OPS, POS)	3.81 (V), 5.10 (A), 3.81 (S/Ps)	3.22	*********
Hurst et al., 2020	USA	133 (133)	54 (54)	52 (52)	COVID-19 (confirmed)	Yes (remdesivir)	Yes (no)	Yes (NPS)	3.77 (V), 15.3 (P), 3.88 (S/Ps), 21.6 (As)	1.38	********
Iwasaki et al., 2020	Japan	5 (5)	0	0	COVID-19 (confirmed)	N/A	Yes (no)	Yes (NPS)	2.53 (V), 3.37 (A), 4.12 (S/Ps)	0.05	****
Kawasuji et al., 2020	Japan	16 (16)	-	-	COVID-19 (confirmed)	Yes (antivirals, antibiotics – specifics not reported)	Yes (no)	Yes (NPS)	3.15 (V)	0.18	*****
L'Huillier et al., 2020	Switzerland	23 (23)	23 (23)	0	COVID-19 (confirmed)	N/A	Yes (no)	Yes (NPS)	2.91 (V), 14.7 (P), 3.73 (S/Ps)	0.24	********
Lavezzo et al., 2020	Italy	103 (110)	2 (3)	49 (49)	COVID-19 (confirmed)	N/A	Yes (yes)	Yes (NPS, OPS)	3.77 (V), 5.03 (A), 11.57 (P), 3.57 (S/Ps), 21.8 (As)	1.14	*******
Lennon et al., 2020	USA	2200 (2,200)	18 (18)	2200 (2200^#^)	COVID-19 (confirmed)	N/A	No (yes)	Yes (NPS)	3.88 (V), 5.20 (A), 24.0 (As)	22.84	*********
Lucas et al., 2020	USA	24 (33)	0	0	COVID-19 (confirmed)	Yes (tocilizumab for moderate and severe patients, glucocorticoid and vasopressor for severe patients)	Yes (yes)	Yes (NPS)	3.51 (V), 4.69 (A), 4.08 (S/Ps)	0.34	*******
Mitjà et al., 2020	Spain	148 (296)	0	0	COVID-19 (confirmed)	N/A	No (no)	Yes (NPS)	3.81 (V), 5.10 (A), 3.93 (S/Ps)	3.07	*********
Pan et al., 2020	China	75 (104)	-	0	COVID-19 (confirmed)	N/A	Yes (no)	Yes (OPS) No (Spu)	3.45 (V), 2.50 (S/Ps)	1.24	****
Peng et al., 2020	China	6 (6)	0	0	COVID-19 (confirmed)	Yes (arbidol, lopinavir, ritonavir, interferon alfa-2b inhalation)	Yes (no)	Yes (OPS)	3.03 (V), 4.05 (A), 4.02 (S/Ps)	0.06	********
Perera et al., 2020	China	- (36)	0	-	COVID-19 (confirmed)	Yes lopinavir-ritonavir alone, combination lopinavir-ritonavir and ribavirin, ribavirin and β interferon, β interferon alone, combination ribavirin, β interferon, and tocilizumab, and corticosteroid	Yes (no)	Yes (NPA, NPS, OPS, Spu)	3.23 (V), 4.32 (A)	0.39	****
Shi et al., 2020	China	103 (103)	0	0	COVID-19 (confirmed)	No (samples drawn before antivirals given)	Yes (no)	Yes (NPS, OPS)	3.87 (V), 5.18 (A), 4.34 (S/Ps)	1.07	*****
Shrestha et al., 2020	USA	171 (171)	0	0	COVID-19 (confirmed)	Yes (indicated no hydroxychloroquine or other COVID-19-related treatments were used)	Yes (no)	Yes (NPS)	3.79 (V), 5.07 (A), 3.86 (S/Ps)	1.78	*******
To et al., 2020	China	23 (51)	0	0	COVID-19 (confirmed)	N/A	Yes (yes)	Yes (ETA, POS)	3.37 (V), 4.51 (A), 3.25 (S/Ps)	0.53	*********
van Kampen et al., 2021	The Netherlands	- (154)	0	0	COVID-19 (confirmed)	Yes (lopinavir-ritonavir with or without ribavirin and/or interferon beta 1b)	Yes (yes)	Yes (NPS, Spu)	3.80 (V), 5.09 (A), 4.10 (S/Ps)	1.60	********
Vetter et al., 2020	Switzerland	5 (63)	0	0	COVID-19 (confirmed)	Yes (paracetamol, alfuzosin, ibuprofen, enoxaparin, amoxicillin clarithromycin, piperacillin, tazobactam, lopinavir, ritonavir, folic acid)	Yes (yes)	Yes (NPS, OPS)	3.68 (V), 4.93 (A), 4.14 (S/Ps)	0.65	*********
Wölfel et al., 2020	Germany	9 (136)	0	1 (4)	COVID-19 (confirmed)	N/A	Yes (yes)	Yes (NPS, OPS) No (Spu)	3.76 (V), 5.03 (A), 3.93 (S/Ps)	1.38	*******
Wyllie et al., 2020	USA	40 (42)	-	9 (9)	COVID-19 (confirmed)	N/A	Yes (yes)	Yes (NPS)	3.55 (V), 4.75 (A), 4.00 (S/Ps), 18.3 (As)	0.44	*******
Xu et al., 2020	China	7 (14)	7 (14)	1 (1)	COVID-19 (confirmed)	Yes (α-interferon oral spray, azithromycin)	Yes (no)	Yes (NPS)	3.40 (V). 17.3 (P), 4.1 (S/Ps)	0.15	********
Yonker et al., 2020	USA	17 (17)	14 (14)	0	COVID-19 (confirmed)	N/A	Yes (no)	Yes (NPS)	2.58 (V), 9.79 (P), 3.28 (S/Ps)	0.18	******
Zhang et al., 2020b	China	9 (9)	0	0	COVID-19 (confirmed)	N/A	Yes (no)	Yes (NPS, OPS)	2.97 (V), 3.97 (A), 3.68 (S/Ps)	0.09	********
Zheng et al., 2020	China	- (19)	0	0	COVID-19 (confirmed)	Yes (gamma globulin, glucocorticoids, antibiotics, antiviral combination of interferon α inhalation, lopinavir-ritonavir combination, arbidol, favipiravir, and darunavir-cobicistat)	Yes (no)	Yes (POS, Spu)	3.66 (V), 4.90 (A), 4.23 (S/Ps)	0.20	*******
Zou et al., 2020	China	14 (55)	0	1 (4)	COVID-19 (confirmed)	N/A	Yes (no)	Yes (NPS, OPS)	3.64 (V), 4.87 (A), 3.65 (S/Ps)	0.57	*******
Chen et al., 2006	China	154 (154^#^)	0	0	SARS (confirmed)	N/A	Yes (no)	Yes (NPS)	14.0 (V)	1.59	********
Chu et al., 2004**	China	11 (11)	0	0	SARS (confirmed)	Yes (control group: ribavirin, hydrocortisone, methylprednisolone)	Yes (yes)	Yes (NPS)	8.6 (V)	0.11	*********
Chu et al., 2005	China	57 (57)	0	0	SARS (confirmed)	N/A	Yes (yes)	Yes (NPA)	13.3 (V)	0.59	*********
Cheng et al., 2004	China	59 (59)	0	0	SARS (confirmed)	Yes (amoxicillin-clavulanate, azithromycin, levofloxacin, ribavirin, hydrocortisone, prednisolone, methylprednisolone)	Yes (yes)	Yes (NPA)	13.4 (V)	0.61	*********
Hung et al., 2004	China	60 (60)	0	0	SARS (confirmed)	N/A	No (yes)	Yes (NPA)	13.5 (V)	0.62	*******
Peiris et al., 2003**	China	14 (42)	0	0	SARS (confirmed)	Yes (ribavirin, hydrocortisone, prednisolone, methylprednisolone)	Yes (no)	Yes (NPA)	13.4 (V)	0.44	********
Poon et al., 2003	China	40 (40)	0	0	SARS (confirmed)	N/A	No (yes)	Yes (NPA)	11.3 (V)	0.42	*****
Poon et al., 2004	China	- (43)	0	0	SARS (confirmed)	N/A	No (yes)	Yes (NPA)	12.5 (V)	0.45	*******
Rodrigues Guimarães Alves et al., 2020	Brazil	86 (86)	-	15 (15)	A(H1N1)pdm09 (confirmed)	Yes (oseltamivir)	No (yes)	Yes (NPA, NPS, OPS)	3.7 (V)	0.89	*****
Chan et al., 2011	China	58 (58)	0	0	A(H1N1)pdm09 (confirmed)	Yes (oseltamivir, zanamivir, peramivir)	Yes (no)	Yes (NPA, NPS, OPS)	3.7 (V)	0.60	******
Cheng et al., 2010	China	60 (60)	-	0	A(H1N1)pdm09 (confirmed)	No (pretreatment samples)	No (no)	Yes (NPA)	3.7 (V)	0.62	******
Cowling et al., 2010	China	45 (54)	22 (31)	0	A(H1N1)pdm09 (confirmed)	Yes (oseltamivir)	Yes (yes)	Yes (NPS, OPS)	3.7 (V)	0.56	*********
Duchamp et al., 2010	France	209 (209)	209 (209)	0	A(H1N1)pdm09 (confirmed)	Yes (oseltamivir, zanamivir)	No (yes)	Yes (NPS)	3.8 (V)	2.17	*****
Esposito et al., 2011	Italy	74 (282)	74 (282)	0	A(H1N1)pdm09 (confirmed)	No	Yes (yes)	Yes (NPS)	3.8 (V)	2.93	*******
Hung et al., 2010	China	87 (87)	-	0	A(H1N1)pdm09 (confirmed)	Yes (oseltamivir)	Yes (no)	Yes (NPA, NPS)	3.8 (V)	0.90	******
Ip et al., 2016	China	17 (20)	7 (-)	0	A(H1N1)pdm09 (confirmed)	N/A	Yes (no)	Yes (NPS, OPS)	3.6 (V)	0.21	*******
Ito et al., 2012	Japan	34 (34)	-	0	A(H1N1)pdm09 (confirmed)	No (pretreatment samples)	Yes (yes)	Yes (NPS)	3.7 (V)	0.35	*****
Killingley et al., 2010	United Kingdom	12 (21)	-	0	A(H1N1)pdm09 (confirmed)	Yes (oseltamivir)	Yes (yes)	Yes (NPS)	3.5 (V)	0.22	********
Launes et al., 2012	Spain	47 (47)	47 (47)	0	A(H1N1)pdm09 (confirmed)	No (pretreatment samples)	No (no)	Yes (NPA)	3.7 (V)	0.49	*******
Lee et al., 2011a	China	48 (48)	0	0	A(H1N1)pdm09 (confirmed)	No (pretreatment samples)	No (no)	Yes (NPA)	3.7 (V)	0.50	********
Lee et al., 2011a	Singapore	578 (578)	231 (231)	0	A(H1N1)pdm09 (confirmed)	No (pretreatment samples)	No (no)	Yes (NPS)	3.8 (V)	6.00	*********
Li et al., 2010a	China	581 (581)	522 (522)	0	A(H1N1)pdm09 (confirmed)	Yes (oseltamivir)	No (no)	Yes (OPS)	3.8 (V)	6.03	********
Li et al., 2010b	China	27 (59)	-	0	A(H1N1)pdm09 (confirmed)	No (control group no treatment)	No (no)	Yes (NPA, NPS, OPS)	3.7 (V)	0.61	*******
Loeb et al., 2012	Canada	97 (218)	-	- (17)	A(H1N1)pdm09 (confirmed)	No	No (no)	Yes (NPS)	3.8 (V)	2.26	*******
Lu et al., 2012	China	13 (25)	-	0	A(H1N1)pdm09 (confirmed)	Yes (oseltamivir, zanamivir)	Yes (no)	Yes (NPS)	3.5 (V)	0.26	*******
Meschi et al., 2011	Italy	533 (533)	0	0	A(H1N1)pdm09 (confirmed)	No (pretreatment samples)	No (no)	Yes (NPS)	3.8 (V)	0.92	*********
Ngaosuwankul et al., 2010	China	12 (33)	-	0	A(H1N1)pdm09 (confirmed)	No (pretreatment samples)	No (yes)	Yes (NPA, NPS, OPS)	3.6 (V)	0.34	******
Rath et al., 2012	Germany	27 (41)	27 (41)	0	A(H1N1)pdm09 (confirmed)	Yes (oseltamivir)	Yes (yes)	Yes (NPS)	3.7 (V)	0.43	*********
Suess et al., 2010	Germany	51 (129)	12 (-)	1 (1)	A(H1N1)pdm09 (confirmed)	Yes (oseltamivir)	No (no)	Yes (NPA, NPS, OPS)	3.8 (V)	1.34	********
Thai et al., 2014	Vietnam	33 (123)	16 (-)	5 (28)	A(H1N1)pdm09 (confirmed)	Yes (oseltamivir)	Yes (yes)	Yes (NPS)	3.8 (V)	1.28	*********
To et al., 2010a	China	50 (50)	0	0	A(H1N1)pdm09 (confirmed)	Yes (oseltamivir, zanamivir, inotropes)	No (no)	Yes (NPA, NPS)	3.6 (V)	0.52	******
To et al., 2010b	China	22 (22)	-	0	A(H1N1)pdm09 (confirmed)	No (pretreatment samples)	No (no)	Yes (NPA, NPS, OPS)	3.4 (V)	0.23	*****
Watanabe et al., 2011	Japan	251 (251)	251 (251)	0	A(H1N1)pdm09 (confirmed)	No (pretreatment samples)	No (yes)	Yes (NPA)	3.8 (V)	2.61	**********
Wu et al., 2012	China	64 (89)	-	0	A(H1N1)pdm09 (confirmed)	Yes (oseltamivir)	No (yes)	Yes (NPS)	3.7 (V)	5.53	*******
Yang et al., 2011	China	251 (251)	-	0	A(H1N1)pdm09 (confirmed)	N/A	No (yes)	Yes (OPS)	3.8 (V)	6.57	*****

^*^Data shown as ‘-' were not obtained from the paper or authors. ^†^Responses of ‘N/A’ indicate that no details were reported on treatment for COVID-19 in the study.

^‡^For studies reporting specimen measurements as individual sample data (either in numerical or graphical formats), the sample data was extracted for analysis. ^§^Specimen measurements were converted to rVLs based on the dilution factor for specimens in transport media.

^||^Abbreviations for random-effects meta-analyses: virus meta-analysis (V), adult subgroup (A), pediatric subgroup (P), symptomatic/presymptomatic subgroup (S/Ps) and asymptomatic subgroup (As). ^¶^The hybrid JBI critical appraisal checklist was used, with more stars indicating lower risk of bias. Studies were considered to have low risk of bias if they met the majority of the items (≥6/10 items). Results from each study are shown in Appendix 1—table 2.

^#^For these studies, 2147 (Lennon et al., 2020) and 134 (Chen et al., 2006) individual specimen measurements were obtained for the individual sample datasets. ^**^For Chu et al., 2004, only specimen measurements at 20 DFSO were collected as 5–15 DFSO were specimens reported in Peiris et al., 2003.

NPS: nasopharyngeal swab; OPS, oropharyngeal swab; Spu: sputum; POS, posterior oropharyngeal saliva; NPA: nasopharyngeal aspirate; ETA: endotracheal aspirate; DFSO: days from symptom onset; rVL: respiratory viral load.

**Appendix 1—table 2.** Assessment of risk of bias based on the hybrid JBI critical appraisal checklist.

	Checklist items*									
**Study**	**1**	**2**	**3**	**4**	**5**	**6**	**7**	**8**	**9**	**10**
Argyropoulos et al., 2020	Y	Y	Y	Y	N	Y	Y	N	Y	Y
Baggio et al., 2020	Y	Y	Y	Y	N	Y	Y	N	N	Y
Fajnzylber et al., 2020	Y	Y	Y	Y	N	Y	Y	Y	N	Y
Hung et al., 2020	N	Y	N	Y	N	Y	Y	N	Y	Y
Hung et al., 2020	Y	Y	Y	Y	N	Y	Y	N	Y	Y
Hung et al., 2020	Y	Y	Y	Y	Y	Y	Y	N	Y	Y
Hung et al., 2020	Y	Y	Y	Y	N	Y	Y	N	Y	Y
Iwasaki et al., 2020	Y	N	U	U	N	Y	Y	N	N	Y
Kawasuji et al., 2020	Y	Y	U	U	N	Y	Y	N	N	Y
L'Huillier et al., 2020	Y	Y	Y	Y	N	Y	Y	N	Y	Y
Lavezzo et al., 2020	Y	Y	N	Y	N	Y	Y	N	Y	Y
Lennon et al., 2020	Y	Y	Y	Y	Y	Y	Y	N	Y	Y
Lucas et al., 2020	Y	Y	U	U	N	Y	Y	Y	Y	Y
Mitjà et al., 2020	Y	Y	Y	Y	Y	Y	Y	N	Y	Y
Pan et al., 2020	Y	N	U	U	N	Y	Y	N	N	Y
Peng et al., 2020	Y	Y	Y	Y	N	Y	Y	N	Y	Y
Perera et al., 2020	Y	N	U	U	N	Y	Y	N	N	Y
Shi et al., 2020	Y	Y	U	U	N	Y	Y	N	N	Y
Shrestha et al., 2020	N	Y	Y	Y	N	Y	Y	N	Y	Y
To et al., 2020	Y	Y	Y	Y	N	Y	Y	Y	Y	Y
van Kampen et al., 2021	Y	Y	Y	Y	N	Y	Y	N	Y	Y
Vetter et al., 2020	Y	Y	Y	Y	N	Y	Y	Y	Y	Y
Wölfel et al., 2020	Y	Y	N	U	N	Y	Y	Y	Y	Y
Wyllie et al., 2020	Y	Y	U	Y	N	Y	Y	Y	N	Y
Xu et al., 2020	Y	Y	Y	Y	N	Y	Y	N	Y	Y
Yonker et al., 2020	Y	Y	N	U	N	Y	Y	N	Y	Y
Zhang et al., 2020b	Y	Y	Y	Y	N	Y	Y	N	Y	Y
Zheng et al., 2020	Y	Y	Y	Y	N	Y	Y	N	N	Y
Zou et al., 2020	N	Y	Y	Y	N	Y	Y	N	Y	Y
Chen et al., 2006	Y	Y	Y	Y	N	Y	Y	N	Y	Y
Chu et al., 2004	Y	Y	Y	Y	N	Y	Y	Y	Y	Y
Chu et al., 2005	Y	Y	Y	Y	N	Y	Y	Y	Y	Y
Cheng et al., 2004	Y	Y	Y	Y	N	Y	Y	Y	Y	Y
Hung et al., 2004	Y	Y	U	U	N	Y	Y	Y	Y	Y
Peiris et al., 2003	Y	Y	Y	Y	N	Y	Y	N	Y	Y
Poon et al., 2003	Y	N	U	U	N	Y	Y	Y	N	Y
Poon et al., 2004	Y	N	Y	Y	N	Y	Y	Y	N	Y
Rodrigues Guimarães Alves et al., 2020	Y	N	U	U	N	Y	Y	Y	N	Y
Chan et al., 2011	Y	Y	U	U	N	Y	Y	N	Y	Y
Cheng et al., 2010	Y	N	Y	Y	N	Y	Y	N	N	Y
Cowling et al., 2010	Y	Y	Y	Y	N	Y	Y	Y	Y	Y
Duchamp et al., 2010	Y	Y	U	U	N	Y	Y	Y	N	N
Esposito et al., 2011	Y	Y	Y	U	N	Y	Y	Y	N	Y
Hung et al., 2010	N	Y	Y	Y	N	Y	Y	N	N	Y
Ip et al., 2016	Y	Y	Y	U	N	Y	Y	N	Y	Y
Ito et al., 2012	Y	N	U	U	N	Y	Y	Y	N	Y
Killingley et al., 2010	Y	Y	Y	Y	N	Y	Y	Y	N	Y
Launes et al., 2012	Y	Y	Y	Y	N	Y	Y	N	N	Y
Lee et al., 2011a	Y	Y	Y	Y	N	Y	Y	N	Y	Y
Lee et al., 2011a	Y	Y	Y	Y	Y	Y	Y	N	Y	Y
Li et al., 2010a	Y	Y	Y	Y	N	Y	Y	N	Y	Y
Li et al., 2010b	Y	Y	Y	Y	N	Y	Y	N	N	Y
Loeb et al., 2012	Y	Y	Y	Y	N	Y	Y	N	N	Y
Lu et al., 2012	Y	Y	Y	Y	N	Y	Y	N	N	Y
Meschi et al., 2011	Y	Y	Y	Y	Y	Y	Y	N	Y	Y
Ngaosuwankul et al., 2010	Y	Y	U	U	N	Y	Y	Y	N	Y
Rath et al., 2012	Y	Y	Y	Y	N	Y	Y	Y	Y	Y
Suess et al., 2010	Y	Y	Y	Y	N	Y	Y	N	Y	Y
Thai et al., 2014	Y	Y	Y	Y	N	Y	Y	Y	Y	Y
To et al., 2010a	Y	Y	U	U	N	Y	Y	N	Y	Y
To et al., 2010b	Y	Y	U	U	N	Y	Y	N	N	Y
Watanabe et al., 2011	Y	Y	Y	Y	Y	Y	Y	Y	Y	Y
Wu et al., 2012	Y	Y	Y	U	N	Y	Y	Y	N	Y
Yang et al., 2011	Y	N	U	U	Y	Y	Y	N	N	Y

*Descriptions of each item are included in the hybrid JBI critical appraisal checklist (Appendix). Y (green), U (yellow) and N (red) represent yes, unclear and no, respectively.

## References

[bib1] Adam DC, Wu P, Wong JY, Lau EHY, Tsang TK, Cauchemez S, Leung GM, Cowling BJ (2020). Clustering and superspreading potential of SARS-CoV-2 infections in Hong Kong. Nature Medicine.

[bib2] Argyropoulos KV, Serrano A, Hu J, Black M, Feng X, Shen G, Call M, Kim MJ, Lytle A, Belovarac B, Vougiouklakis T, Lin LH, Moran U, Heguy A, Troxel A, Snuderl M, Osman I, Cotzia P, Jour G (2020). Association of initial viral load in severe acute respiratory syndrome coronavirus 2 (SARS-CoV-2) Patients with outcome and symptoms. The American Journal of Pathology.

[bib3] Arons MM, Hatfield KM, Reddy SC, Kimball A, James A, Jacobs JR, Taylor J, Spicer K, Bardossy AC, Oakley LP, Tanwar S, Dyal JW, Harney J, Chisty Z, Bell JM, Methner M, Paul P, Carlson CM, McLaughlin HP, Thornburg N, Tong S, Tamin A, Tao Y, Uehara A, Harcourt J, Clark S, Brostrom-Smith C, Page LC, Kay M, Lewis J, Montgomery P, Stone ND, Clark TA, Honein MA, Duchin JS, Jernigan JA (2020). Presymptomatic SARS-CoV-2 infections and transmission in a skilled nursing facility. New England Journal of Medicine.

[bib4] Asadi S, Wexler AS, Cappa CD, Barreda S, Bouvier NM, Ristenpart WD (2020). Effect of voicing and articulation manner on aerosol particle emission during human speech. PLOS ONE.

[bib5] Baccam P, Beauchemin C, Macken CA, Hayden FG, Perelson AS (2006). Kinetics of influenza A virus infection in humans. Journal of Virology.

[bib6] Baggio S, L'Huillier AG, Yerly S, Bellon M, Wagner N, Rohr M, Huttner A, Blanchard-Rohner G, Loevy N, Kaiser L, Jacquerioz F, Eckerle I (2020). SARS-CoV-2 viral load in the upper respiratory tract of children and adults with early acute COVID-19. Clinical Infectious Diseases.

[bib7] Bi Q, Wu Y, Mei S, Ye C, Zou X, Zhang Z, Liu X, Wei L, Truelove SA, Zhang T, Gao W, Cheng C, Tang X, Wu X, Wu Y, Sun B, Huang S, Sun Y, Zhang J, Ma T, Lessler J, Feng T (2020). Epidemiology and transmission of COVID-19 in 391 cases and 1286 of their close contacts in Shenzhen, China: a retrospective cohort study. The Lancet Infectious Diseases.

[bib8] Bird JC, de Ruiter R, Courbin L, Stone HA (2010). Daughter bubble cascades produced by folding of ruptured thin films. Nature.

[bib9] Brugger J, Althaus CL (2020). Transmission of and susceptibility to seasonal influenza in Switzerland from 2003 to 2015. Epidemics.

[bib10] Chan PK, Lee N, Joynt GM, Choi KW, Cheung JL, Yeung AC, Lam P, Wong R, Leung BW, So HY, Lam WY, Hui DC (2011). Clinical and virological course of infection with haemagglutinin D222G mutant strain of 2009 pandemic influenza A (H1N1) virus. Journal of Clinical Virology.

[bib11] Chen WJ, Yang JY, Lin JH, Fann CS, Osyetrov V, King CC, Chen YM, Chang HL, Kuo HW, Liao F, Ho MS (2006). Nasopharyngeal shedding of severe acute respiratory syndrome-associated coronavirus is associated with genetic polymorphisms. Clinical Infectious Diseases.

[bib12] Chen PZ (2020). GitHub.

[bib13] Chen PZ, Bobrovitz N, Premji Z, Koopmans M, Fisman DN, Gu F (2021). SARS-CoV-2 shedding dynamics across the respiratory tract, sex, and disease severity for adult and pediatric COVID-19. medRxiv.

[bib14] Cheng VC, Hung IF, Tang BS, Chu CM, Wong MM, Chan KH, Wu AK, Tse DM, Chan KS, Zheng BJ, Peiris JS, Sung JJ, Yuen KY (2004). Viral replication in the nasopharynx is associated with diarrhea in patients with severe acute respiratory syndrome. Clinical Infectious Diseases.

[bib15] Cheng PK, Wong KK, Mak GC, Wong AH, Ng AY, Chow SY, Lam RK, Lau CS, Ng KC, Lim W (2010). Performance of laboratory diagnostics for the detection of influenza A(H1N1)v virus as correlated with the time after symptom onset and viral load. Journal of Clinical Virology.

[bib16] Cheng HY, Jian SW, Liu DP, Ng TC, Huang WT, Lin HH, Taiwan COVID-19 Outbreak Investigation Team (2020). Contact tracing assessment of COVID-19 transmission dynamics in Taiwan and risk at different exposure periods before and after symptom onset. JAMA Internal Medicine.

[bib17] Chu CM, Cheng VC, Hung IF, Wong MM, Chan KH, Chan KS, Kao RY, Poon LL, Wong CL, Guan Y, Peiris JS, Yuen KY, HKU/UCH SARS Study Group (2004). Role of lopinavir/ritonavir in the treatment of SARS: initial virological and clinical findings. Thorax.

[bib18] Chu CM, Cheng VC, Hung IF, Chan KS, Tang BS, Tsang TH, Chan KH, Yuen KY (2005). Viral load distribution in SARS outbreak. Emerging Infectious Diseases.

[bib19] Chu DK, Akl EA, Duda S, Solo K, Yaacoub S, Schünemann HJ, Chu DK, Akl EA, El-harakeh A, Bognanni A, Lotfi T, Loeb M, Hajizadeh A, Bak A, Izcovich A, Cuello-Garcia CA, Chen C, Harris DJ, Borowiack E, Chamseddine F, Schünemann F, Morgano GP, Muti Schünemann GEU, Chen G, Zhao H, Neumann I, Chan J, Khabsa J, Hneiny L, Harrison L, Smith M, Rizk N, Giorgi Rossi P, AbiHanna P, El-khoury R, Stalteri R, Baldeh T, Piggott T, Zhang Y, Saad Z, Khamis A, Reinap M, Duda S, Solo K, Yaacoub S, Schünemann HJ (2020). Physical distancing, face masks, and eye protection to prevent person-to-person transmission of SARS-CoV-2 and COVID-19: a systematic review and meta-analysis. The Lancet.

[bib20] Cowling BJ, Chan KH, Fang VJ, Lau LLH, So HC, Fung ROP, Ma ESK, Kwong ASK, Chan C-W, Tsui WWS, Ngai H-Y, Chu DWS, Lee PWY, Chiu M-C, Leung GM, Peiris JSM (2010). Comparative epidemiology of pandemic and seasonal influenza A in households. New England Journal of Medicine.

[bib21] Cowling BJ, Ip DKM, Fang VJ, Suntarattiwong P, Olsen SJ, Levy J, Uyeki TM, Leung GM, Malik Peiris JS, Chotpitayasunondh T, Nishiura H, Mark Simmerman J (2013). Aerosol transmission is an important mode of influenza A virus spread. Nature Communications.

[bib22] Dong E, Du H, Gardner L (2020). An interactive web-based dashboard to track COVID-19 in real time. The Lancet Infectious Diseases.

[bib23] Du Z, Xu X, Wu Y, Wang L, Cowling BJ, Meyers LA (2020). Serial interval of COVID-19 among publicly reported confirmed cases. Emerging Infectious Diseases.

[bib24] Duchamp MB, Casalegno JS, Gillet Y, Frobert E, Bernard E, Escuret V, Billaud G, Valette M, Javouhey E, Lina B, Floret D, Morfin F (2010). Pandemic A(H1N1)2009 influenza virus detection by real time RT-PCR: is viral quantification useful?. Clinical Microbiology and Infection.

[bib25] Endo A, Abbott S, Kucharski AJ, Funk S, Centre for the Mathematical Modelling of Infectious Diseases COVID-19 Working Group (2020). Estimating the overdispersion in COVID-19 transmission using outbreak sizes outside China. Wellcome Open Research.

[bib26] Esposito S, Daleno C, Baldanti F, Scala A, Campanini G, Taroni F, Fossali E, Pelucchi C, Principi N (2011). Viral shedding in children infected by pandemic A/H1N1/2009 influenza virus. Virology Journal.

[bib27] Fabian P, McDevitt JJ, DeHaan WH, Fung ROP, Cowling BJ, Chan KH, Leung GM, Milton DK (2008). Influenza virus in human exhaled breath: an observational study. PLOS ONE.

[bib28] Fajnzylber J, Regan J, Coxen K, Corry H, Wong C, Rosenthal A, Worrall D, Giguel F, Piechocka-Trocha A, Atyeo C, Fischinger S, Chan A, Flaherty KT, Hall K, Dougan M, Ryan ET, Gillespie E, Chishti R, Li Y, Jilg N, Hanidziar D, Baron RM, Baden L, Tsibris AM, Armstrong KA, Kuritzkes DR, Alter G, Walker BD, Yu X, Li JZ (2020). SARS-CoV-2 viral load is associated with increased disease severity and mortality. Nature Communications.

[bib29] Fears AC, Klimstra WB, Duprex P, Hartman A, Weaver SC, Plante KC, Roy CJ (2020). Comparative dynamic aerosol efficiencies of three emergent coronaviruses and the unusual persistence of SARS-CoV-2 in aerosol suspensions. medRxiv.

[bib30] Guan WJ, Ni ZY, Hu Y, Liang WH, Ou CQ, He JX, Liu L, Shan H, Lei CL, Hui DSC, Du B, Li LJ, Zeng G, Yuen KY, Chen RC, Tang CL, Wang T, Chen PY, Xiang J, Li SY, Wang JL, Liang ZJ, Peng YX, Wei L, Liu Y, Hu YH, Peng P, Wang JM, Liu JY, Chen Z, Li G, Zheng ZJ, Qiu SQ, Luo J, Ye CJ, Zhu SY, Zhong NS, China Medical Treatment Expert Group for Covid-19 (2020). Clinical characteristics of coronavirus disease 2019 in China. New England Journal of Medicine.

[bib31] Hamner L, Dubbel P, Capron I, Ross A, Jordan A, Lee J, Lynn J, Ball A, Narwal S, Russell S, Patrick D, Leibrand H (2020). High SARS-CoV-2 attack rate following exposure at a choir practice - Skagit county, Washington, march 2020. MMWR. Morbidity and Mortality Weekly Report.

[bib32] Han MS, Seong MW, Heo EY, Park JH, Kim N, Shin S, Cho SI, Park SS, Choi EH (2020a). Sequential analysis of viral load in a neonate and her mother infected with severe acute respiratory syndrome coronavirus 2. Clinical Infectious Diseases.

[bib33] Han MS, Seong MW, Kim N, Shin S, Cho SI, Park H, Kim TS, Park SS, Choi EH (2020b). Viral RNA load in mildly symptomatic and asymptomatic children with COVID-19, Seoul, South Korea. Emerging Infectious Diseases.

[bib34] Han MS, Byun JH, Cho Y, Rim JH (2021). RT-PCR for SARS-CoV-2: quantitative versus qualitative. The Lancet Infectious Diseases.

[bib35] Hao X, Cheng S, Wu D, Wu T, Lin X, Wang C (2020). Reconstruction of the full transmission dynamics of COVID-19 in Wuhan. Nature.

[bib36] He X, Lau EHY, Wu P, Deng X, Wang J, Hao X, Lau YC, Wong JY, Guan Y, Tan X, Mo X, Chen Y, Liao B, Chen W, Hu F, Zhang Q, Zhong M, Wu Y, Zhao L, Zhang F, Cowling BJ, Li F, Leung GM (2020). Temporal dynamics in viral shedding and transmissibility of COVID-19. Nature Medicine.

[bib37] Higgins JPT, Thomas J, Chandler J, Cumpston M, Li T, Page MJ, Welch VA (2019). Cochrane Handbook for Systematic Reviews of Interventions, Cochrane Book Series.

[bib38] Hung IFN, Cheng VCC, Wu AKL, Tang BSF, Chan KH, Chu CM, Wong MML, Hui WT, Poon LLM, Tse DMW, Chan KS, Woo PCY, Lau SKP, Peiris JSM, Yuen KY (2004). Viral loads in clinical specimens and SARS manifestations. Emerging Infectious Diseases.

[bib39] Hung IF, To KK, Lee CK, Lin CK, Chan JF, Tse H, Cheng VC, Chen H, Ho PL, Tse CW, Ng TK, Que TL, Chan KH, Yuen KY (2010). Effect of clinical and virological parameters on the level of neutralizing antibody against pandemic influenza A virus H1N1 2009. Clinical Infectious Diseases.

[bib40] Hung IF-N, Lung K-C, Tso EY-K, Liu R, Chung TW-H, Chu M-Y, Ng Y-Y, Lo J, Chan J, Tam AR, Shum H-P, Chan V, Wu AK-L, Sin K-M, Leung W-S, Law W-L, Lung DC, Sin S, Yeung P, Yip CC-Y, Zhang RR, Fung AY-F, Yan EY-W, Leung K-H, Ip JD, Chu AW-H, Chan W-M, Ng AC-K, Lee R, Fung K, Yeung A, Wu T-C, Chan JW-M, Yan W-W, Chan W-M, Chan JF-W, Lie AK-W, Tsang OT-Y, Cheng VC-C, Que T-L, Lau C-S, Chan K-H, To KK-W, Yuen K-Y (2020). Triple combination of interferon beta-1b, lopinavir–ritonavir, and ribavirin in the treatment of patients admitted to hospital with COVID-19: an open-label, randomised, phase 2 trial. The Lancet.

[bib41] Hurst JH, Heston SM, Chambers HN, Cunningham HM, Price MJ, Suarez L, Kelly MS (2020). SARS-CoV-2 infections among children in the biospecimens from respiratory virus-exposed kids (BRAVE kids) study. medRxiv.

[bib42] Ikeda H, Nakaoka S, de Boer RJ, Morita S, Misawa N, Koyanagi Y, Aihara K, Sato K, Iwami S (2016). Quantifying the effect of vpu on the promotion of HIV-1 replication in the humanized mouse model. Retrovirology.

[bib43] Ip DKM, Lau LLH, Chan KH, Fang VJ, Leung GM, Peiris MJS, Cowling BJ (2016). The dynamic relationship between clinical symptomatology and viral shedding in naturally acquired seasonal and pandemic influenza virus infections. Clinical Infectious Diseases.

[bib44] Ip DK, Lau LL, Leung NH, Fang VJ, Chan KH, Chu DK, Leung GM, Peiris JS, Uyeki TM, Cowling BJ (2017). Viral shedding and transmission potential of asymptomatic and paucisymptomatic influenza virus infections in the community. Clinical Infectious Diseases.

[bib45] Ito M, Nukuzuma S, Sugie M, Yoshioka M, Kon-no M, Yasutake H, Umegaki Y, Ishikawa Y, Yano T, Ihara T (2012). Detection of pandemic influenza A (H1N1) 2009 virus RNA by real-time reverse transcription polymerase chain reaction. Pediatrics International.

[bib46] Iwasaki S, Fujisawa S, Nakakubo S, Kamada K, Yamashita Y, Fukumoto T, Sato K, Oguri S, Taki K, Senjo H, Sugita J, Hayasaka K, Konno S, Nishida M, Teshima T (2020). Comparison of SARS-CoV-2 detection in nasopharyngeal swab and saliva. Journal of Infection.

[bib47] Jeong HW, Kim SM, Kim HS, Kim YI, Kim JH, Cho JY, Kim SH, Kang H, Kim SG, Park SJ, Kim EH, Choi YK (2020). Viable SARS-CoV-2 in various specimens from COVID-19 patients. Clinical Microbiology and Infection.

[bib48] Johnson GR, Morawska L, Ristovski ZD, Hargreaves M, Mengersen K, Chao CYH, Wan MP, Li Y, Xie X, Katoshevski D, Corbett S (2011). Modality of human expired aerosol size distributions. Journal of Aerosol Science.

[bib49] Johnson GR, Morawska L (2009). The mechanism of breath aerosol formation. Journal of Aerosol Medicine and Pulmonary Drug Delivery.

[bib50] Johnston KM, Lakzadeh P, Donato BMK, Szabo SM (2019). Methods of sample size calculation in descriptive retrospective burden of illness studies. BMC Medical Research Methodology.

[bib51] Kawasuji H, Takegoshi Y, Kaneda M, Ueno A, Miyajima Y, Kawago K, Yamamoto Y (2020). Viral load dynamics in transmissible symptomatic patients with COVID-19. medRxiv.

[bib52] Killingley B, Greatorex J, Cauchemez S, Enstone JE, Curran M, Read RC, Lim WS, Hayward A, Nicholson KG, Nguyen-Van-Tam JS (2010). Virus shedding and environmental deposition of novel A (H1N1) pandemic influenza virus: interim findings. Health Technology Assessment.

[bib53] L'Huillier AG, Torriani G, Pigny F, Kaiser L, Eckerle I (2020). Culture-Competent SARS-CoV-2 in nasopharynx of symptomatic neonates, children, and adolescents. Emerging Infectious Diseases.

[bib54] Lau LLH, Cowling BJ, Fang VJ, Chan K, Lau EHY, Lipsitch M, Cheng CKY, Houck PM, Uyeki TM, Peiris JSM, Leung GM (2010). Viral shedding and clinical illness in naturally acquired influenza virus infections. The Journal of Infectious Diseases.

[bib55] Launes C, Garcia-Garcia JJ, Jordan I, Selva L, Rello J, Muñoz-Almagro C (2012). Viral load at diagnosis and influenza A H1N1 (2009) disease severity in children. Influenza and Other Respiratory Viruses.

[bib56] Lavezzo E, Franchin E, Ciavarella C, Cuomo-Dannenburg G, Barzon L, Del Vecchio C, Rossi L, Manganelli R, Loregian A, Navarin N, Abate D, Sciro M, Merigliano S, De Canale E, Vanuzzo MC, Besutti V, Saluzzo F, Onelia F, Pacenti M, Parisi SG, Carretta G, Donato D, Flor L, Cocchio S, Masi G, Sperduti A, Cattarino L, Salvador R, Nicoletti M, Caldart F, Castelli G, Nieddu E, Labella B, Fava L, Drigo M, Gaythorpe KAM, Brazzale AR, Toppo S, Trevisan M, Baldo V, Donnelly CA, Ferguson NM, Dorigatti I, Crisanti A, Imperial College COVID-19 Response Team (2020). Suppression of a SARS-CoV-2 outbreak in the italian municipality of vo'. Nature.

[bib57] Laxminarayan R, Wahl B, Dudala SR, Gopal K, Mohan B C, Neelima S, Jawahar Reddy KS, Radhakrishnan J, Lewnard JA (2020). Epidemiology and transmission dynamics of COVID-19 in two indian states. Science.

[bib58] Lednicky JA, Lauzardo M, Fan ZH, Jutla A, Tilly TB, Gangwar M, Usmani M, Shankar SN, Mohamed K, Eiguren-Fernandez A, Stephenson CJ, Alam MM, Elbadry MA, Loeb JC, Subramaniam K, Waltzek TB, Cherabuddi K, Morris JG, Wu CY (2020). Viable SARS-CoV-2 in the air of a hospital room with COVID-19 patients. International Journal of Infectious Diseases.

[bib59] Lee CK, Lee HK, Loh TP, Lai FY, Tambyah PA, Chiu L, Koay ES, Tang JW (2011a). Comparison of pandemic (H1N1) 2009 and seasonal influenza viral loads, Singapore. Emerging Infectious Diseases.

[bib60] Lee N, Chan PK, Wong CK, Wong KT, Choi KW, Joynt GM, Lam P, Chan MC, Wong BC, Lui GC, Sin WW, Wong RY, Lam WY, Yeung AC, Leung TF, So HY, Yu AW, Sung JJ, Hui DS (2011b). Viral clearance and inflammatory response patterns in adults hospitalized for pandemic 2009 influenza A(H1N1) virus pneumonia. Antiviral Therapy.

[bib61] Lee EC, Wada NI, Grabowski MK, Gurley ES, Lessler J (2020). The engines of SARS-CoV-2 spread. Science.

[bib62] Lennon NJ, Bhattacharyya RP, Mina MJ, Rehm HL, Hung DT, Smole S, Gabriel SB (2020). Omparison of viral levels in individuals with or without symptoms at time of COVID-19 testing among 32,480 residents and staff of nursing homes and assisted living facilities in Massachusetts. medRxiv.

[bib63] Li CC, Wang L, Eng HL, You HL, Chang LS, Tang KS, Lin YJ, Kuo HC, Lee IK, Liu JW, Huang EY, Yang KD (2010a). Correlation of pandemic (H1N1) 2009 viral load with disease severity and prolonged viral shedding in children. Emerging Infectious Diseases.

[bib64] Li IW, Hung IF, To KK, Chan KH, Wong SS, Chan JF, Cheng VC, Tsang OT, Lai ST, Lau YL, Yuen KY (2010b). The natural viral load profile of patients with pandemic 2009 influenza A(H1N1) and the effect of oseltamivir treatment. Chest.

[bib65] Li Q, Guan X, Wu P, Wang X, Zhou L, Tong Y, Ren R, Leung KSM, Lau EHY, Wong JY, Xing X, Xiang N, Wu Y, Li C, Chen Q, Li D, Liu T, Zhao J, Liu M, Tu W, Chen C, Jin L, Yang R, Wang Q, Zhou S, Wang R, Liu H, Luo Y, Liu Y, Shao G, Li H, Tao Z, Yang Y, Deng Z, Liu B, Ma Z, Zhang Y, Shi G, Lam TTY, Wu JT, Gao GF, Cowling BJ, Yang B, Leung GM, Feng Z (2020a). Early transmission dynamics in Wuhan, China, of novel Coronavirus-Infected pneumonia. New England Journal of Medicine.

[bib66] Li R, Pei S, Chen B, Song Y, Zhang T, Yang W, Shaman J (2020b). Substantial undocumented infection facilitates the rapid dissemination of novel coronavirus (SARS-CoV-2). Science.

[bib67] Lieber C, Melekidis S, Koch R, Bauer HJ (2021). Insights into the evaporation characteristics of saliva droplets and aerosols: levitation experiments and numerical modeling. Journal of Aerosol Science.

[bib68] Liu L, Li Y, Nielsen PV, Wei J, Jensen RL (2017a). Short-range airborne transmission of expiratory droplets between two people. Indoor Air.

[bib69] Liu L, Wei J, Li Y, Ooi A (2017b). Evaporation and dispersion of respiratory droplets from coughing. Indoor Air.

[bib70] Lloyd-Smith JO, Schreiber SJ, Kopp PE, Getz WM (2005). Superspreading and the effect of individual variation on disease emergence. Nature.

[bib71] Loeb M, Singh PK, Fox J, Russell ML, Pabbaraju K, Zarra D, Wong S, Neupane B, Singh P, Webby R, Fonseca K (2012). Longitudinal study of influenza molecular viral shedding in hutterite communities. Journal of Infectious Diseases.

[bib72] Lu PX, Deng YY, Yang GL, Liu WL, Liu YX, Huang H, Wang YX (2012). Relationship between respiratory viral load and lung lesion severity: a study in 24 cases of pandemic H1N1 2009 influenza A pneumonia. Journal of Thoracic Disease.

[bib73] Lu J, Gu J, Li K, Xu C, Su W, Lai Z, Zhou D, Yu C, Xu B, Yang Z (2020a). COVID-19 outbreak associated with air conditioning in restaurant, Guangzhou, China, 2020. Emerging Infectious Diseases.

[bib74] Lu X, Zhang L, Du H, Zhang J, Li YY, Qu J, Zhang W, Wang Y, Bao S, Li Y, Wu C, Liu H, Liu D, Shao J, Peng X, Yang Y, Liu Z, Xiang Y, Zhang F, Silva RM, Pinkerton KE, Shen K, Xiao H, Xu S, Wong GWK (2020b). SARS-CoV-2 infection in children. New England Journal of Medicine.

[bib75] Lucas C, Wong P, Klein J, Castro TBR, Silva J, Sundaram M, Ellingson MK, Mao T, Oh JE, Israelow B, Takahashi T, Tokuyama M, Lu P, Venkataraman A, Park A, Mohanty S, Wang H, Wyllie AL, Vogels CBF, Earnest R, Lapidus S, Ott IM, Moore AJ, Muenker MC, Fournier JB, Campbell M, Odio CD, Casanovas-Massana A, Herbst R, Shaw AC, Medzhitov R, Schulz WL, Grubaugh ND, Dela Cruz C, Farhadian S, Ko AI, Omer SB, Iwasaki A, Yale IMPACT Team (2020). Longitudinal analyses reveal immunological misfiring in severe COVID-19. Nature.

[bib76] Luo L, Liu D, Liao X, Wu X, Jing Q, Zheng J, Liu F, Yang S, Bi H, Li Z, Liu J, Song W, Zhu W, Wang Z, Zhang X, Huang Q, Chen P, Liu H, Cheng X, Cai M, Yang P, Yang X, Han Z, Tang J, Ma Y, Mao C (2020). Contact settings and risk for transmission in 3410 close contacts of patients with COVID-19 in Guangzhou, China : a prospective cohort study. Annals of Internal Medicine.

[bib77] Ma J, Qi X, Chen H, Li X, Zhang Z, Wang H, Yao M (2020). COVID-19 patients in earlier stages exhaled millions of SARS-CoV-2 per hour. Clinical Infectious Diseases.

[bib78] Meschi S, Selleri M, Lalle E, Bordi L, Valli MB, Ferraro F, Ippolito G, Petrosillo N, Lauria FN, Capobianchi MR (2011). Duration of viral shedding in hospitalized patients infected with pandemic H1N1. BMC Infectious Diseases.

[bib79] Mitjà O, Corbacho-Monné M, Ubals M, Tebe C, Peñafiel J, Tobias A, Ballana E, Alemany A, Riera-Martí N, Pérez CA, Suñer C, Laporte P, Admella P, Mitjà J, Clua M, Bertran L, Sarquella M, Gavilán S, Ara J, Argimon JM, Casabona J, Cuatrecasas G, Cañadas P, Elizalde-Torrent A, Fabregat R, Farré M, Forcada A, Flores-Mateo G, Muntada E, Nadal N, Narejos S, Gil-Ortega AN, Prat N, Puig J, Quiñones C, Reyes-Ureña J, Ramírez-Viaplana F, Ruiz L, Riveira-Muñoz E, Sierra A, Velasco C, Vivanco-Hidalgo RM, Sentís A, G-Beiras C, Clotet B, Vall-Mayans M, BCN PEP-CoV-2 RESEARCH GROUP (2020). Hydroxychloroquine for early treatment of adults with mild Covid-19: a randomized-controlled trial. Clinical Infectious Diseases.

[bib80] Moher D, Liberati A, Tetzlaff J, Altman DG, PRISMA Group (2009). Preferred reporting items for systematic reviews and meta-analyses: the PRISMA statement. PLOS Medicine.

[bib81] Moola S, Munn Z, Tufanaru C, Aromataris E, Sears K, Sfetcu R, Mu P, Aromataris E, Munn Z (2020). Chapter 7: systematic reviews of etiology and risk. Joanna Briggs Institute Reviewer's Manual. the Joanna Briggs Institute.

[bib82] Morawska L, Johnson GR, Ristovski ZD, Hargreaves M, Mengersen K, Corbett S, Chao CYH, Li Y, Katoshevski D (2009). Size distribution and sites of origin of droplets expelled from the human respiratory tract during expiratory activities. Journal of Aerosol Science.

[bib83] Morris DH, Yinda KC, Gamble A, Rossine FW, Huang Q, Bushmaker T, Lloyd-Smith JO (2020). Mechanistic theory predicts the effects of temperature and humidity on inactivation of SARS-CoV-2 and other enveloped viruses. bioRxiv.

[bib84] Munn Z, Moola S, Lisy K, Riitano D, Tufanaru C (2015). Methodological guidance for systematic reviews of observational epidemiological studies reporting prevalence and cumulative incidence data. International Journal of Evidence-Based Healthcare.

[bib85] Munn Z, Barker TH, Moola S, Tufanaru C, Stern C, McArthur A, Aromataris E (2019). Methodological quality of case series studies: an introduction to the JBI critical appraisal tool. JBI Database of Systematic Reviews and Implementation Reports.

[bib86] Ngaosuwankul N, Noisumdaeng P, Komolsiri P, Pooruk P, Chokephaibulkit K, Chotpitayasunondh T, Sangsajja C, Chuchottaworn C, Farrar J, Puthavathana P (2010). Influenza A viral loads in respiratory samples collected from patients infected with pandemic H1N1, seasonal H1N1 and H3N2 viruses. Virology Journal.

[bib87] Pan Y, Zhang D, Yang P, Poon LLM, Wang Q (2020). Viral load of SARS-CoV-2 in clinical samples. The Lancet Infectious Diseases.

[bib88] Park SY, Kim YM, Yi S, Lee S, Na BJ, Kim CB, Kim JI, Kim HS, Kim YB, Park Y, Huh IS, Kim HK, Yoon HJ, Jang H, Kim K, Chang Y, Kim I, Lee H, Gwack J, Kim SS, Kim M, Kweon S, Choe YJ, Park O, Park YJ, Jeong EK (2020). Coronavirus disease outbreak in call center, South Korea. Emerging Infectious Diseases.

[bib89] Peiris JSM, Chu CM, Cheng VCC, Chan KS, Hung IFN, Poon LLM, Law KI, Tang BSF, Hon TYW, Chan CS, Chan KH, Ng JSC, Zheng BJ, Ng WL, Lai RWM, Guan Y, Yuen KY (2003). Clinical progression and viral load in a community outbreak of coronavirus-associated SARS pneumonia: a prospective study. The Lancet.

[bib90] Peng L, Liu J, Xu W, Luo Q, Chen D, Lei Z, Huang Z, Li X, Deng K, Lin B, Gao Z (2020). SARS-CoV-2 can be detected in urine, blood, anal swabs, and oropharyngeal swabs specimens. Journal of Medical Virology.

[bib91] Perera R, Tso E, Tsang OTY, Tsang DNC, Fung K, Leung YWY, Chin AWH, Chu DKW, Cheng SMS, Poon LLM, Chuang VWM, Peiris M (2020). SARS-CoV-2 virus culture and subgenomic RNA for respiratory specimens from patients with mild coronavirus disease. Emerging Infectious Diseases.

[bib92] Pitzer VE, Leung GM, Lipsitch M (2007). Estimating variability in the transmission of severe acute respiratory syndrome to household contacts in Hong Kong, China. American Journal of Epidemiology.

[bib93] Poon LL, Chan KH, Wong OK, Yam WC, Yuen KY, Guan Y, Lo YM, Peiris JS (2003). Early diagnosis of SARS coronavirus infection by real time RT-PCR. Journal of Clinical Virology.

[bib94] Poon LL, Chan KH, Wong OK, Cheung TK, Ng I, Zheng B, Seto WH, Yuen KY, Guan Y, Peiris JS (2004). Detection of SARS coronavirus in patients with severe acute respiratory syndrome by conventional and real-time quantitative reverse transcription-PCR assays. Clinical Chemistry.

[bib95] Prather KA, Marr LC, Schooley RT, McDiarmid MA, Wilson ME, Milton DK (2020). Airborne transmission of SARS-CoV-2. Science.

[bib96] Rath B, von Kleist M, Tief F, Karsch K, Tuerk E, Muehlhans S, Louis F, Skopnik H, Schweiger B, Duwe S (2012). Virus load kinetics and resistance development during oseltamivir treatment in infants and children infected with influenza A(H1N1) 2009 and influenza B viruses. Pediatric Infectious Disease Journal.

[bib97] Riou J, Althaus CL (2020). Pattern of early human-to-human transmission of wuhan 2019 novel coronavirus (2019-nCoV), December 2019 to January 2020. Eurosurveillance.

[bib98] Roberts MG, Nishiura H (2011). Early estimation of the reproduction number in the presence of imported cases: pandemic influenza H1N1-2009 in New Zealand. PLOS ONE.

[bib99] Rodrigues Guimarães Alves V, Perosa AH, de Souza Luna LK, Cruz JS, Conte DD, Bellei N (2020). Influenza A(H1N1)pdm09 infection and viral load analysis in patients with different clinical presentations. Memórias Do Instituto Oswaldo Cruz.

[bib100] Roy CJ, Milton DK (2004). Airborne transmission of communicable infection — The Elusive Pathway. New England Journal of Medicine.

[bib101] Schijven J, Vermeulen LC, Swart A, Meijer A, Duizer E, de Roda Husman AM (2020). Exposure assessment for airborne transmission of SARS-CoV-2 via breathing, speaking, coughing and sneezing. medRxiv.

[bib102] Shi F, Wu T, Zhu X, Ge Y, Zeng X, Chi Y, Du X, Zhu L, Zhu F, Zhu B, Cui L, Wu B (2020). Association of viral load with serum biomakers among COVID-19 cases. Virology.

[bib103] Shrestha NK, Marco Canosa F, Nowacki AS, Procop GW, Vogel S, Fraser TG, Erzurum SC, Terpeluk P, Gordon SM (2020). Distribution of Transmission Potential During Nonsevere COVID-19 Illness. Clinical Infectious Diseases.

[bib104] Suess T, Buchholz U, Dupke S, Grunow R, an der Heiden M, Heider A, Biere B, Schweiger B, Haas W, Krause G, Robert Koch Institute Shedding Investigation Group (2010). Shedding and transmission of novel influenza virus A/H1N1 infection in households--Germany, 2009. American Journal of Epidemiology.

[bib105] Tariq A, Lee Y, Roosa K, Blumberg S, Yan P, Ma S, Chowell G (2020). Real-time monitoring the transmission potential of COVID-19 in Singapore, March 2020. BMC Medicine.

[bib106] Team CC-R, CDC COVID-19 Response Team (2020). Coronavirus disease 2019 in children - United states, February 12-April 2, 2020. MMWR. Morbidity and Mortality Weekly Report.

[bib107] Thai PQ, Mai LQ, Welkers MRA, Hang NLK, Thanh LT, Dung VTV, Yen NTT, Duong TN, Hoa LNM, Thoang DD, Trang HTH, de Jong MD, Wertheim H, Hien NT, Horby P, Fox A (2014). Pandemic H1N1 virus transmission and shedding dynamics in index case households of a prospective Vietnamese cohort. Journal of Infection.

[bib108] To KK, Chan K-H, Li IWS, Tsang T-Y, Tse H, Chan JFW, Hung IFN, Lai S-T, Leung C-W, Kwan Y-W, Lau Y-L, Ng T-K, Cheng VCC, Peiris JSM, Yuen K-Y (2010a). Viral load in patients infected with pandemic H1N1 2009 influenza A virus. Journal of Medical Virology.

[bib109] To KK, Hung IF, Li IW, Lee KL, Koo CK, Yan WW, Liu R, Ho KY, Chu KH, Watt CL, Luk WK, Lai KY, Chow FL, Mok T, Buckley T, Chan JF, Wong SS, Zheng B, Chen H, Lau CC, Tse H, Cheng VC, Chan KH, Yuen KY (2010b). Delayed clearance of viral load and marked cytokine activation in severe cases of pandemic H1N1 2009 influenza virus infection. Clinical Infectious Diseases : An Official Publication of the Infectious Diseases Society of America.

[bib110] To KK, Tsang OT-Y, Leung W-S, Tam AR, Wu T-C, Lung DC, Yip CC-Y, Cai J-P, Chan JM-C, Chik TS-H, Lau DP-L, Choi CY-C, Chen L-L, Chan W-M, Chan K-H, Ip JD, Ng AC-K, Poon RW-S, Luo C-T, Cheng VC-C, Chan JF-W, Hung IF-N, Chen Z, Chen H, Yuen K-Y (2020). Temporal profiles of viral load in posterior oropharyngeal saliva samples and serum antibody responses during infection by SARS-CoV-2: an observational cohort study. The Lancet Infectious Diseases.

[bib111] van Kampen JJA, van de Vijver DAMC, Fraaij PLA, Haagmans BL, Lamers MM, Okba N, van den Akker JPC, Endeman H, Gommers D, Cornelissen JJ, Hoek RAS, van der Eerden MM, Hesselink DA, Metselaar HJ, Verbon A, de Steenwinkel JEM, Aron GI, van Gorp ECM, van Boheemen S, Voermans JC, Boucher CAB, Molenkamp R, Koopmans MPG, Geurtsvankessel C, van der Eijk AA (2021). Duration and key determinants of infectious virus shedding in hospitalized patients with coronavirus disease-2019 (COVID-19). Nature Communications.

[bib112] Vetter P, Eberhardt CS, Meyer B, Martinez Murillo PA, Torriani G, Pigny F, Lemeille S, Cordey S, Laubscher F, Vu DL, Calame A, Schibler M, Jacquerioz F, Blanchard-Rohner G, Siegrist CA, Kaiser L, Didierlaurent AM, Eckerle I (2020). Daily viral kinetics and innate and adaptive immune response assessment in COVID-19: a case series. mSphere.

[bib113] Wan X, Wang W, Liu J, Tong T (2014). Estimating the sample mean and standard deviation from the sample size, median, range and/or interquartile range. BMC Medical Research Methodology.

[bib114] Warnke P, Warning L, Podbielski A (2014). Some Are More Equal - A Comparative Study on Swab Uptake and Release of Bacterial Suspensions. PLOS ONE.

[bib115] Watanabe M, Nukuzuma S, Ito M, Ihara T (2011). Viral load and rapid diagnostic test in patients with pandemic H1N1 2009. Pediatrics International.

[bib116] Wei WE, Li Z, Chiew CJ, Yong SE, Toh MP, Lee VJ (2020). Presymptomatic Transmission of SARS-CoV-2 â€” Singapore, January 23â€“March 16, 2020. MMWR. Morbidity and Mortality Weekly Report.

[bib117] Wei J, Li Y (2015). Enhanced spread of expiratory droplets by turbulence in a cough jet. Building and Environment.

[bib118] Wölfel R, Corman VM, Guggemos W, Seilmaier M, Zange S, Müller MA, Niemeyer D, Jones TC, Vollmar P, Rothe C, Hoelscher M, Bleicker T, Brünink S, Schneider J, Ehmann R, Zwirglmaier K, Drosten C, Wendtner C (2020). Virological assessment of hospitalized patients with COVID-2019. Nature.

[bib119] Wu U-I, Wang J-T, Chen Y-C, Chang S-C (2012). Severity of pandemic H1N1 2009 influenza virus infection may not be directly correlated with initial viral load in upper respiratory tract. Influenza and Other Respiratory Viruses.

[bib120] Wyllie AL, Fournier J, Casanovas-Massana A, Campbell M, Tokuyama M, Vijayakumar P, Warren JL, Geng B, Muenker MC, Moore AJ, Vogels CBF, Petrone ME, Ott IM, Lu P, Venkataraman A, Lu-Culligan A, Klein J, Earnest R, Simonov M, Datta R, Handoko R, Naushad N, Sewanan LR, Valdez J, White EB, Lapidus S, Kalinich CC, Jiang X, Kim DJ, Kudo E, Linehan M, Mao T, Moriyama M, Oh JE, Park A, Silva J, Song E, Takahashi T, Taura M, Weizman O-E, Wong P, Yang Y, Bermejo S, Odio CD, Omer SB, Dela Cruz CS, Farhadian S, Martinello RA, Iwasaki A, Grubaugh ND, Ko AI (2020). Saliva or Nasopharyngeal Swab Specimens for Detection of SARS-CoV-2. New England Journal of Medicine.

[bib121] Xu Y, Li X, Zhu B, Liang H, Fang C, Gong Y, Guo Q, Sun X, Zhao D, Shen J, Zhang H, Liu H, Xia H, Tang J, Zhang K, Gong S (2020). Characteristics of pediatric SARS-CoV-2 infection and potential evidence for persistent fecal viral shedding. Nature Medicine.

[bib122] Yan J, Grantham M, Pantelic J, Bueno de Mesquita PJ, Albert B, Liu F, Ehrman S, Milton DK, Consortium E, EMIT Consortium (2018). Infectious virus in exhaled breath of symptomatic seasonal influenza cases from a college community. PNAS.

[bib123] Yang J-R, Lo J, Ho Y-L, Wu H-S, Liu M-T (2011). Pandemic H1N1 and seasonal H3N2 influenza infection in the human population show different distributions of viral loads, which substantially affect the performance of rapid influenza tests. Virus Research.

[bib124] Yonker LM, Neilan AM, Bartsch Y, Patel AB, Regan J, Arya P, Gootkind E, Park G, Hardcastle M, St. John A, Appleman L, Chiu ML, Fialkowski A, De la Flor D, Lima R, Bordt EA, Yockey LJ, D'Avino P, Fischinger S, Shui JE, Lerou PH, Bonventre JV, Yu XG, Ryan ET, Bassett IV, Irimia D, Edlow AG, Alter G, Li JZ, Fasano A (2020). Pediatric Severe Acute Respiratory Syndrome Coronavirus 2 (SARS-CoV-2): Clinical Presentation, Infectivity, and Immune Responses. The Journal of Pediatrics.

[bib125] Yu ITS, Li Y, Wong TW, Tam W, Chan AT, Lee JHW, Leung DYC, Ho T (2004). Evidence of Airborne Transmission of the Severe Acute Respiratory Syndrome Virus. New England Journal of Medicine.

[bib126] Zhang N, Gong Y, Meng F, Shi Y, Wang J, Mao P, Chuai X, Bi Y, Yang P, Wang F (2020a). Comparative study on virus shedding patterns in nasopharyngeal and fecal specimens of COVID-19 patients. Science China Life Sciences.

[bib127] Zhang Y, Li Y, Wang L, Li M, Zhou X (2020b). Evaluating Transmission Heterogeneity and Super-Spreading Event of COVID-19 in a Metropolis of China. International Journal of Environmental Research and Public Health.

[bib128] Zheng S, Fan J, Yu F, Feng B, Lou B, Zou Q, Xie G, Lin S, Wang R, Yang X, Chen W, Wang Q, Zhang D, Liu Y, Gong R, Ma Z, Lu S, Xiao Y, Gu Y, Zhang J, Yao H, Xu K, Lu X, Wei G, Zhou J, Fang Q, Cai H, Qiu Y, Sheng J, Chen Y, Liang T (2020). Viral load dynamics and disease severity in patients infected with SARS-CoV-2 in Zhejiang Province, China, January-March 2020: retrospective cohort study. BMJ.

[bib129] Zou L, Ruan F, Huang M, Liang L, Huang H, Hong Z, Yu J, Kang M, Song Y, Xia J, Guo Q, Song T, He J, Yen H-L, Peiris M, Wu J (2020). SARS-CoV-2 Viral Load in Upper Respiratory Specimens of Infected Patients. New England Journal of Medicine.

