# Supplementary material for: Heterogeneity in transmissibility and shedding SARS-CoV-2 via droplets and aerosols
Source: eLife. 2021 Apr 16;10:e65774. doi: 10.7554/eLife.65774 (PMC8139838; doi:10.7554/eLife.65774)
Supplement: Figure 1—source data 1. [file elife-65774-fig1-data1.docx]

Figure 1—Source Data 1. Search strategy used for MEDLINE.

| Database: **Ovid MEDLINE® and Epub Ahead of Print, In-Process & Other Non-Indexed Citations and Daily**1946 to 7 August 2020 | |
| --- | --- |
| **#** | **Searches** |
| 1 | exp Coronavirus/ |
| 2 | exp Coronavirus Infections/ |
| 3 | exp Betacoronavirus/ |
| 4 | (coronavirus* or corona virus* or betacoronavirus* or OC43 or NL63 or 229E or HKU1 or HcoV* or ncov* or covid* or sars-cov* or sarscov* or Sars-coronavirus* or Severe Acute Respiratory Syndrome* or sudden acute respiratory syndrome*).tw,kf. |
| 5 | (2019nCov* or 2019-novel CoV or corona or covid19 or ((novel or new or nouveau) adj2 (CoV or Pandemi*))).tw,kf. |
| 6 | (pneumonia.tw,kf. Or exp pneumonia/) and (Wuhan or Hubei).tw,kf. |
| 7 | COVID-19.rx,px,ox. Or severe acute respiratory syndrome coronavirus 2.os. |
| 8 | exp Influenza A Virus, H1N1 Subtype/ |
| 9 | (“A/H1N1*” or H1N1* or pdm09 or ((influenza or virus or pandemic) adj4 “2009”) or influenza A or swine flu).tw,kf. |
| 10 | 1 or 2 or 3 or 4 or 5 or 6 or 7 or 8 or 9 |
| 11 | ((respiratory adj3 (specimen* or sample* or swab*)) or sputum or nares or endotrachea* or endotrache* or endotra* or ((nasal or oral* or throat) adj3 (swab* or sample* or smear* or specimen*)) or NPS or OPS or ((endotrachea* or endotracheal*) adj2 aspirat*) or NPA or ETA or (deep adj4 saliva) or POS or “swab sample*” or “flocked swab*”).tw,kf. |
| 12 | Nasal cavity/vi |
| 13 | Sputum/vi |
| 14 | Nasopharynx/vi |
| 15 | Oropharynx/vi |
| 16 | *Saliva/vi |
| 17 | Pharynx/vi |
| 18 | (clinical adj2 (sample* or specimen*)).tw,kf. |
| 19 | (“RT-PCR” or “RTPCR” or “ddPCR” or “polymerase chain reaction”).tw,kf. |
| 20 | Influenza, Human/vi |
| 21 | exp Coronavirus Infections/vi or exp Coronavirus/vi or exp Betacoronavirus/vi |
| 22 | polymerase chain reaction/ or multiplex polymerase chain reaction/ or real-time polymerase chain reaction/ or reverse transcriptase polymerase chain reaction/ |
| 23 | 11 or 12 or 13 or 14 or 15 or 16 or 17 or 18 or 19 or 20 or 21 or 22 |
| 24 | 10 and 23 |
| 25 | (vir* load* or vir* shed* or vir* burden or vir* titer* or vir* titre* or (vir* adj2 count*)).tw,kf. |
| 26 | (((copies or copy) adj2 (ml* or milli* or microl*)) or ((RNA* or vir*) adj2 concentration*)).tw,kf. |
| 27 | ((calibration adj1 curve*) or (standard adj1 curve*)).tw,kf. |
| 28 | ((ct* adj1 value*) or cycle threshold or (copies adj2 test*) or (copy adj2 test*) or ((copy or copies) adj2 number*)).tw,kf. |
| 29 | Viral load/ |
| 30 | Viral shedding/ |
| 31 | (“copy/m*” or “copies/m*” or “copy/test*” or “copies/test*”).tw,kf. |
| 32 | ((test or diagnos*) adj2 sensitiv*).tw,kf. |
| 33 | 25 or 26 or 27 or 28 or 29 or 30 or 31 or 32 |
| 34 | 24 and 33 |
| 35 | animals/ not humans/ |
| 36 | 34 not 35 |
