# Supplementary material for: Heterogeneity in transmissibility and shedding SARS-CoV-2 via droplets and aerosols
Source: eLife. 2021 Apr 16;10:e65774. doi: 10.7554/eLife.65774 (PMC8139838; doi:10.7554/eLife.65774)
Supplement: Figure 1—source data 5. [file elife-65774-fig1-data5.docx]

Figure 1—Source Data 5. Search strategy used for medRxiv and bioRxiv.

| **medRxiv + bioRxiv (via Publish or Perish program)** up to 7 August 2020 |
| --- |
| **Keywords:** (covid OR coronavirus OR ncov OR hcov OR h1n1 OR “swine flu” OR COVID19 or SARS) AND (“copies/ml” OR “copy/ml” OR “viral load” OR “copies/test” OR “copy/test” OR “copies per ml” OR “copy per ml”)  **Publication name:** MedRxiv |
| **Keywords:** (covid OR coronavirus OR ncov OR hcov OR h1n1 OR “swine flu” OR COVID19 or SARS) AND (“copies/ml” OR “copy/ml” OR “viral load” OR “copies/test” OR “copy/test” OR “copies per ml” OR “copy per ml”)  **Publication name:** BioRxiv |
