# Supplementary material for: Heterogeneity in transmissibility and shedding SARS-CoV-2 via droplets and aerosols
Source: eLife. 2021 Apr 16;10:e65774. doi: 10.7554/eLife.65774 (PMC8139838; doi:10.7554/eLife.65774)
Supplement: Figure 4—figure supplement 3—source data 1. [file elife-65774-fig4-figsupp3-data1.docx]

Figure 4—Figure supplement 3. Descriptive parameters for respiratory viral loads based on individual sample data.

|  | | | **Weibull distribution parameters** | | **Respiratory viral load, log_10_ copies/ml** | | | | |
| --- | --- | --- | --- | --- | --- | --- | --- | --- | --- |
| **Category** | ***n*** * **(specimens)** | ***n*** * **(studies)** | **Scale factor**  **(95% CI)** | **Shape factor (95% CI)** | **Mean (95% CI)**^†^ | **SD**^†^ | **80^th^ percentile (95% CI)**^‡^ | **90^th^ percentile (95% CI)**^‡^ | **99^th^ percentile (95% CI)**^‡^ |
| SARS-CoV-2 (overall)^§^ | 3,834 | 26 | 7.01 (6.94-7.08) | 3.47 (3.39-3.56) | 6.29 (6.22-6.35) | 2.04 | 8.04 (7.96-8.11) | 8.91 (8.83-9.00) | 10.88 (10.75-11.01) |
| SARS-CoV-1 (overall)^§^ | 303 | 5 | 6.37 (6.15-6.60) | 3.40 (3.12-3.71) | 5.72 (5.51-5.93) | 1.86 | 7.33 (7.09-7.57) | 8.14 (7.86-8.43) | 9.98 (9.56-10.42) |
| A(H1N1)pdm09 (overall)^§^ | 512 | 10 | 7.39 (7.27-7.51) | 5.43 (5.07-5.81) | 6.81 (6.69-6.94) | 1.45 | 8.07 (7.94-8.20) | 8.62 (8.47-8.76) | 9.79 (9.59-10.00) |
| SARS-CoV-2 (adult)^§^ | 3,575 | 20 | 7.00 (6.93-7.07) | 3.48 (3.39-3.57) | 6.27 (6.21-6.34) | 2.03 | 8.02 (7.95-8.10) | 8.89 (8.81-8.98) | 10.86 (10.72-10.99) |
| SARS-CoV-2 (pediatric)^§^ | 198 | 9 | 7.43 (7.14-7.74) | 3.63 (3.25-4.05) | 6.69 (6.40-6.97) | 2.06 | 8.47 (8.15-8.80) | 9.35 (8.98-9.73) | 11.32 (10.76-11.90) |
| SARS-CoV-2 (symptomatic/presymptomatic)^§^ | 1,574 | 22 | 7.40 (7.30-7.51) | 3.81 (3.67-3.97) | 6.68 (6.58-6.79) | 2.00 | 8.39 (8.28-8.50) | 9.21 (9.09-9.34) | 11.05 (10.86-11.24) |
| SARS-CoV-2 (asymptomatic)^§^ | 2,221 | 7 | 6.72 (6.63-6.81) | 3.33 (3.22-3.44) | 6.01 (5.92-6.09) | 2.01 | 8.04 (7.96-8.11) | 8.91 (8.83-9.00) | 10.88 (10.75-11.01) |
| SARS-CoV-2 (all DFSO)^§^ | 955 | 21 | 7.07 (6.94-7.21) | 3.50 (3.33-3.68) | 6.35 (6.22-6.48) | 2.03 | 8.10 (7.95-8.25) | 8.97 (8.80-9.15) | 10.94 (10.68-11.21) |
| SARS-CoV-2 (-3 DFSO)^\|\|^ | 1 | 1 | - | - | 10.34 | - | - | - | - |
| SARS-CoV-2 (-2 DFSO)^\|\|^ | 3 | 2 | - | - | 4.22 (2.41-6.02) | 1.59 | - | - | - |
| SARS-CoV-2 (-1 DFSO) | 15 | 5 | 6.17 (5.11-7.47) | 2.82 (1.89-4.19) | 5.48 (4.25-6.70) | 2.21 | 7.31 (6.11-8.75) | 8.30 (6.88-10.02) | 10.62 (8.38-13.45) |
| SARS-CoV-2 (0 DFSO) | 50 | 11 | 6.66 (6.13-7.24) | 3.52 (2.87-4.32) | 6.00 (5.49-6.51) | 1.83 | 7.62 (7.05-8.25) | 8.44 (7.78-9.16) | 10.28 (9.30-11.36) |
| SARS-CoV-2 (1 DFSO) | 63 | 11 | 7.86 (7.33-8.43) | 3.71 (3.04-4.53) | 7.08 (6.54-7.63) | 2.22 | 8.94 (8.36-9.55) | 9.84 (9.17-10.56) | 11.86 (10.84-12.99) |
| SARS-CoV-2 (2 DFSO)^¶^ | 71 | 15 | 7.33 (6.84-7.87) | 3.46 (2.85-4.19) | 6.58 (6.07-7.10) | 2.22 | 8.42 (7.87-9.01) | 9.34 (8.69-10.03) | 11.41 (10.39-12.53) |
| SARS-CoV-2 (3 DFSO)^¶^ | 75 | 17 | 7.24 (6.73-7.78) | 3.25 (2.70-3.92) | 6.47 (5.95-6.98) | 2.28 | 8.38 (7.81-8.98) | 9.35 (8.68-10.07) | 11.57 (10.52-12.72) |
| SARS-CoV-2 (4 DFSO)^¶^ | 85 | 17 | 6.83 (6.29-7.41) | 2.75 (2.32-3.27) | 6.06 (5.54-6.58) | 2.44 | 8.12 (7.51-8.77) | 9.25 (8.52-10.03) | 11.90 (10.72-13.20) |
| SARS-CoV-2 (5 DFSO)^¶^ | 93 | 16 | 7.16 (6.69-7.66) | 3.17 (2.69-3.73) | 6.41 (5.95-6.87) | 2.26 | 8.32 (7.80-8.87) | 9.31 (8.70-9.97) | 11.59 (10.63-12.64) |
| SARS-CoV-2 (6 DFSO)^¶^ | 105 | 15 | 6.84 (6.41-7.29) | 3.13 (2.67-3.66) | 6.10 (5.68-6.53) | 2.23 | 7.96 (7.49-8.46) | 8.93 (8.36-9.53) | 11.14 (10.24-12.12) |
| SARS-CoV-2 (7 DFSO)^¶^ | 136 | 20 | 6.59 (6.23-6.97) | 3.11 (2.71-3.56) | 5.90 (5.55-6.26) | 2.12 | 7.68 (7.27-8.11) | 8.62 (8.14-9.13) | 10.77 (10.05-11.60) |
| SARS-CoV-2 (8 DFSO)^¶^ | 123 | 19 | 6.51 (6.12-6.92) | 3.03 (2.62-3.49) | 5.82 (5.44-6.19) | 2.13 | 7.62 (7.18-8.08) | 8.58 (8.06-9.12) | 10.78 (9.96-11.67) |
| SARS-CoV-2 (9 DFSO)^¶^ | 128 | 19 | 6.26 (5.87-6.67) | 2.87 (2.50-3.29) | 5.57 (5.20-5.94) | 2.14 | 7.38 (6.95-7.85) | 8.37 (7.85-8.92) | 10.66 (9.83-11.55) |
| SARS-CoV-2 (10 DFSO)^¶^ | 115 | 18 | 5.71 (5.30-6.16) | 2.55 (2.20-2.95) | 5.09 (4.70-5.48) | 2.14 | 6.89 (6.41-7.40) | 7.93 (7.35-8.56) | 10.41 (9.45-11.47) |

*These two columns summarize the cumulative number of specimens (left) collected from the number of contributing studies (right) for each category in the systematic dataset.

^†^The mean and sample SD were calculated on the entirety of individual sample data for each category. These data were collected from studies clearly reporting data for individual specimens.

^‡^The Weibull quantile distributions were used to determine rVLs at the 80^th^, 90^th^ and 99^th^ cps.

^§^These categories included only rVL data from positive (above the detection limit) assay measurements.

^||^Data for earlier DFSO were excluded from distribution fitting based on limited data, and empty cells were marked with “-“.

^¶^These categories included negative assay measurements (set at the detection limit to estimate rVLs; *N* = 5, 3, 7, 10, 13, 17, 14, 22 and 17 specimens for 2-10 DFSO, respectively) for cases that tested positive at an earlier DFSO.
