# Supplementary material for: Heterogeneity in transmissibility and shedding SARS-CoV-2 via droplets and aerosols
Source: eLife. 2021 Apr 16;10:e65774. doi: 10.7554/eLife.65774 (PMC8139838; doi:10.7554/eLife.65774)
Supplement: Figure 4—figure supplement 4—source data 1. [file elife-65774-fig4-figsupp4-data1.docx]

| **Parameter** | **Description** | **Value (95% CI)** | **Units** |
| --- | --- | --- | --- |
| *β* | Infection rate constant | 3.26 (2.21-4.31) | ×10^-7^ (copies/ml)^-1^ day^-1^ |
| *p* | Cellular shedding rate of virus | 1.33 (0.74-1.93) | copies/ml day^-1^ cell^-1^ |
| *c* | Clearance rate of virus | 3.30 (0.25-6.34) | day^-1^ |
| *t*_1/2_ | Half-life of viral RNA | 5.04 (2.62-66.0) | day |
| *δ* | Clearance rate of infected epithelial cells | 0.71 (0.26-1.15) | day^-1^ |
| *R*_0,c_ | Cellular basic reproductive number | 9.25 | unitless |

Figure 4—Figure supplement 4. Model parameters describing SARS-CoV-2 kinetics during respiratory infection.
